# Supplementary material for: The transcriptional co-repressor Runx1t1 is essential for MYCN-driven neuroblastoma tumorigenesis
Source: Nat Commun. 2024 Jul 11;15:5585. doi: 10.1038/s41467-024-49871-0 (PMC11239676; doi:10.1038/s41467-024-49871-0)

Supplementary Information

The transcriptional co-repressor Runx1t1 is essential for MYCN-driven neuroblastoma tumorigenesis

Jayne E. Murray[1,13], Emanuele Valli[1,13], Giorgio Milazzo[2,13], Chelsea Mayoh[1,3], Andrew J. Gifford[1,3,4], Jamie I. Fletcher[1,3], Chengyuan Xue[1], Nisitha Jayatilleke[1], Firoozeh Salehzadeh[1], Laura D. Gamble[1], Jourdin R. C. Rouaen[1], Daniel R. Carter[1,3,5], Helen Forgham[1], Eric O. Sekyere[1], Joanna Keating[1], Georgina Eden[1], Sophie Allan[1], Stephanie Alfred[1], Frances K. Kusuma[1], Ashleigh Clark[1], Hannah Webber[1], Amanda J. Russell[1,15], Antoine de Weck[1], Benjamin T. Kile[6,7], Martina Santulli[2], Piergiuseppe De Rosa[2], Emmy D. G. Fleuren[1], Weiman Gao[1], Lorna Wilkinson-White[8], Jason K. K. Low[9], Joel P. Mackay[9], Glenn M. Marshall[1,3,10], Douglas J. Hilton[7], Federico M. Giorgi[2], Jan Koster[11], Giovanni Perini[2,14], Michelle Haber[1,4,14], Murray D. Norris[1,12,14,*]

[1]Children's Cancer Institute, Lowy Cancer Centre, UNSW Sydney, Kensington, NSW, 2031, Australia.
[2]Department of Pharmacy and Biotechnology, University of Bologna, 40126 Bologna, Italy.
[3]School of Clinical Medicine, UNSW Sydney, NSW, Australia.
[4]Anatomical Pathology, NSW Health Pathology, Prince of Wales Hospital, Randwick, NSW, Australia.
[5]School of Biomedical Engineering, University of Technology Sydney, Broadway, NSW, Australia.
[6]Monash Biomedicine Discovery Institute, Monash University, Melbourne, Australia.
[7]The Walter and Eliza Hall Institute of Medical Research, Parkville, Victoria, Australia.
[8]Sydney Analytical Core Research Facility, The University of Sydney, Sydney, Australia.
[9]School of Life and Environmental Sciences, The University of Sydney, Sydney Australia.
[10]Kids Cancer Centre, Sydney Children's Hospital, Randwick, NSW, Australia.
[11]Academic Medical Center, University of Amsterdam, Amsterdam, Netherlands.
[12]UNSW Centre for Childhood Cancer Research, UNSW Sydney, NSW, Australia.
[13]These authors contributed equally: Jayne E Murray, Emanuele Valli, Giorgio Milazzo.
[14]These authors jointly supervised this work: Giovanni Perini, Michelle Haber, Murray D Norris.
[15]Present address: Garvan Institute of Medical Research, Darlinghurst, NSW, Australia

***Correspondence Murray Norris, Email: : mnorris@ccia.unsw.edu.au**

Inventory of Supporting Information

Supplementary Figures

1. Supp Fig. 1 ENU mutagenesis identifies Runx1t1 responsible for loss of tumor formation.
2. Supp Fig. 2 Runx1t1 protein translation driven by MYCN…
3. Supp Fig. 3 RUNX1T1 loss leads to downregulation of MYCN dimerization partner MAX
4. Supp Fig. 4 Colony formation and histopathology staining following …
5. Supp Fig. 5 RUNX1T1 forms part of an LCH repressor complex
6. Supp Fig. 6 RUNX1T1 depletion downregulates Hallmark oxidative phosphorylation…
7. Supp Fig. 7 RUNX1T1 loss reduces proliferation in alveolar rhabdomyosarcoma …

Supplementary Tables

1. Supp Table 1 Doxycycline-induced knockdown of RUNX1T1 in KELLY xenografts
2. Supp Table 2 Primers and probes used in real-time PCR analysis for mouse genotyping
3. Supp Table 3 Primers for expression assays
4. Supp Table 4 Primers used to create 5'UTR RUNX1T1 constructs
5. Supp Table 5 Sequence of oligos used to create RUNX1T1 shRNA constructs
6. Supp Table 6 Primers used to create mutant RUNX1T1 con
7. Supp Table 7 Primers for Max ChIP-qPCR assays
8. Supp Table 8 Antibodies used

Supplementary Fig. 1

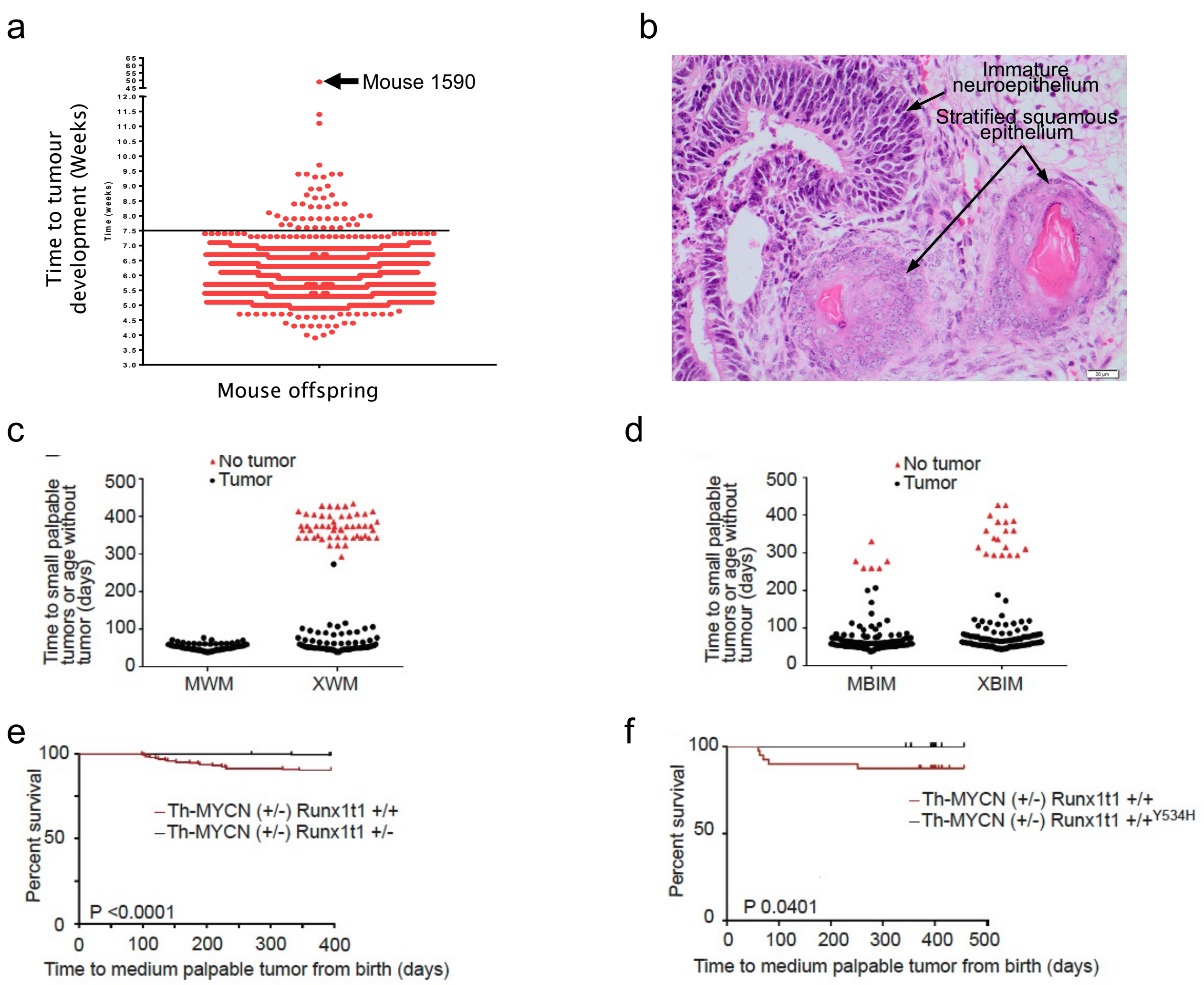

Supplementary Fig. 1: ENU mutagenesis identifies Runx1t1 responsible for loss of tumor formation. (**a**) Tumor delay in offspring of female Th-MYCN mice crossed with ENU-treated male mice (n=1716). (**b**) H&E staining of the tumor from mouse #1590 demonstrating different cell types. (**c**) Backcrossing of the #1590 line to Balb/c mice compared to non-mutated Th-MYCN mice backcrossed in the same fashion. Mice that developed tumors are shown as black circles while those that did not develop tumors are shown as red triangles. MWM are unmutated Th-MYCN mice crossed with Balb/c and then backcrossed to Th-MYCN mice (n=107), and XWM represent #1590 mutated mice crossed in the same way (n=149) (**d**) Backcrossing of the #1590 line to C57BL/6 mice compared to non-mutated Th-MYCN mice backcrossed in the same fashion. Mice that developed tumors are shown as black circles while those that did not develop tumors are shown as red triangles. MBIM are unmutated Th-MYCN mice crossed with C57BL/6 and then backcrossed to Th-MYCN mice (n=115), and XBIM represent #1590 mutated mice crossed in the same way (n=144) (**e**) Kaplan-Meier survival analysis of mice hemizygous for the Th-MYCN transgene and with heterozygous knock-out of Runx1t1 (i.e. Th-MYCN (+/-); Runx1t1 (+/-); black line; n=347) demonstrate significantly decreased tumor incidence by comparison with hemizygous Th-MYCN mice with wild-type Runx1t1 (i.e. Th-MYCN (+/-); Runx1t1 (+/+); red line; n=183; $p<0.0001$ by log-rank test). (**f**) Kaplan-Meier survival curve of mice hemizygous for the Th-MYCN transgene and either wild-type for Runx1t1 (red; n=40) or harboring the Runx1t1 Y534H mutation (black; n=32, $p = 0.0401$ by log-rank test).

Supplementary Fig. 2

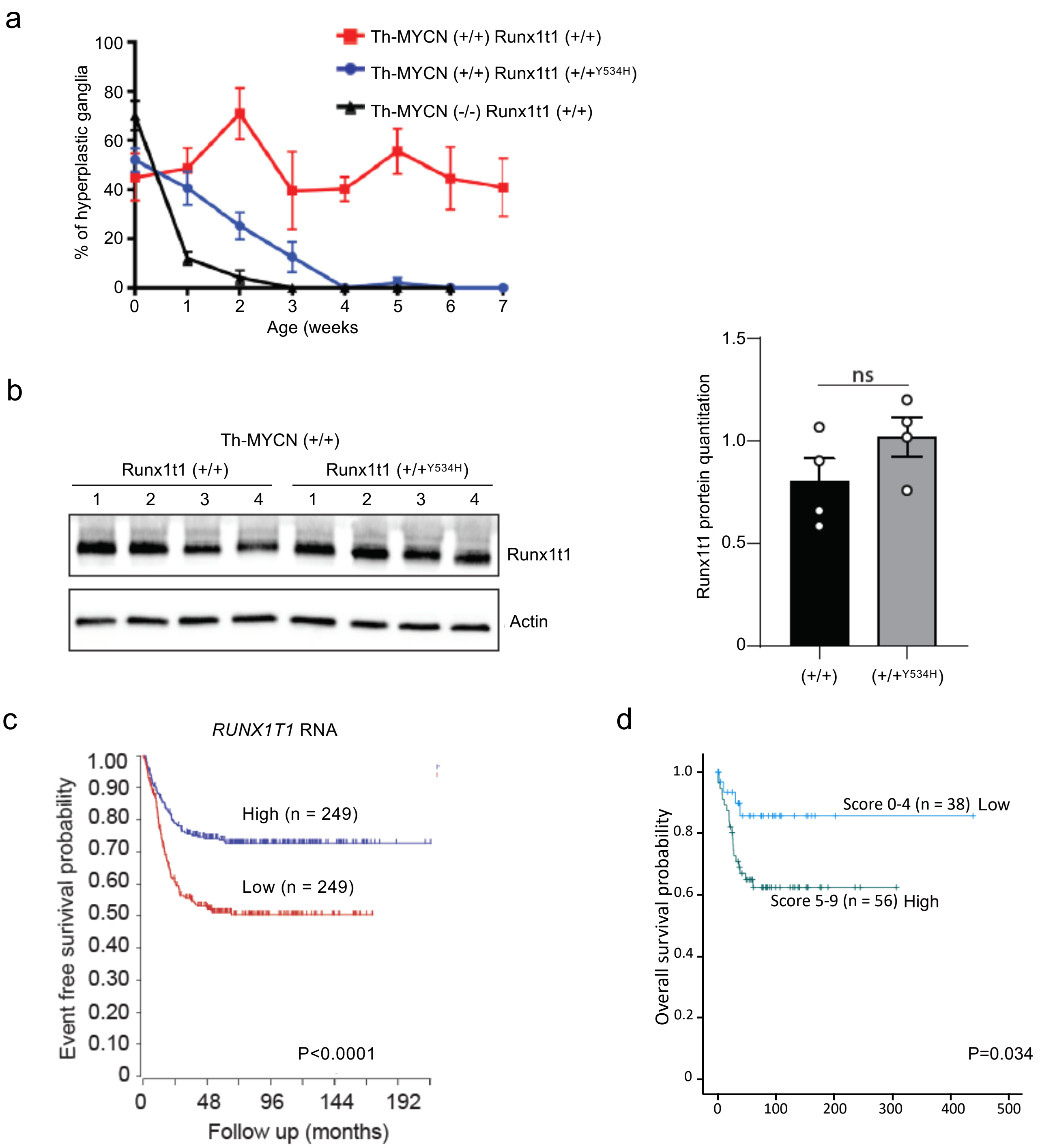

Supplementary Fig. 2: Runx1t1 protein translation driven by MYCN is required to sustain MYCN-mediated hyperplasia. (**a**) The percentage neuroblast hyperplasia scored from homozygous or wild-type Th-MYCN mice, with either wild-type or mutated (Y534H) Runx1t1. Scoring of n≥3 mice was done for each genotype and timepoint. Th-MYCN (+/+) Runx1t1 (+/+) all time points n=4 except day 0 (n=9), 4 weeks (n=3), 5 weeks (n=8), 7 weeks (n=7); Th-MYCN (+/+) Runx1t1 (+/+Y534H) all time points n=5 except day 0, 1 and 2 weeks (n=6), 7 weeks (n=3); Th-MYCN (-/-) Runx1t1 (+/+) all time points n=4 except day 0 (n=10), 1 and 2 weeks (n=6), 5 weeks (n=5). (**b**) Western Blot of Runx1t1 on brain tissue samples obtained at Day 0 from mice homozygous for the Th-MYCN transgene and either wild-type (+/+) or mutant (+/+Y534H) for Runx1t1, demonstrating no significant difference in protein levels. Quantitation of western blot, relative to the Actin control. Values represent means of 4 different brain samples for each genotype ± SEM. Each data point shows the mean value of 3 independent Western blots. Mann Whitney 2-tailed test p=0.2 (**c**) Kaplan-Meier survival analysis of RUNX1T1 expression levels in the publicly available RNAseq dataset of 498 human neuroblastoma samples from the neuroblastoma R2 database. Cohort was cut at the median into high (n=249) and low (n=249) RUNX1T1 expression, p< 0.0001 (log-rank test). (**d**) Kaplan-Meier survival analysis based on RUNX1T1 protein expression in a TMA of primary neuroblastoma (n=77), ganglioneuroblastoma (n=5) and ganglioneuroma (n=12). Samples were grouped based on the RUNX1T1 score 0-4 (n=38) and 4-9 (n=56); (p=0.034).

Supplementary Fig. 3

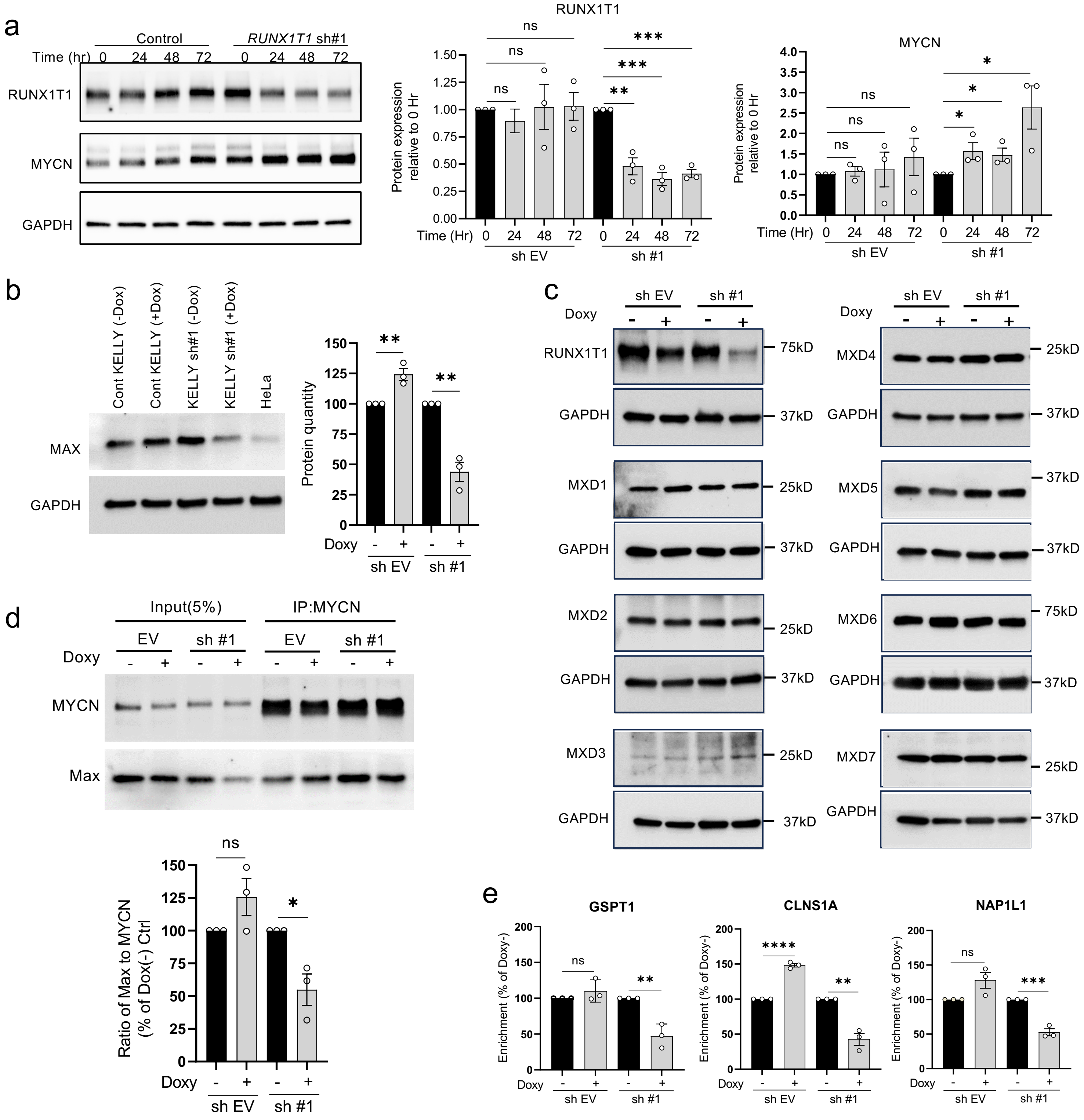

Supplementary Fig. 3: RUNX1T1 loss leads to downregulation of MYCN dimerization partner MAX. (**a**) Following RUNX1T1 shRNA downregulation in KELLY cells, RUNX1T1 and MYCN protein levels were monitored at 24, 48 and 72 hr timepoints. Despite a significant decrease in RUNX1T1 ($p < 0.05$), MYCN protein was significantly increased. Values are mean ± SEM from 3 independent experiments. Two-tailed unpaired t-test (RUNX1T1 shEV 24hr p=0.3973, 48hr p=0.9152, 72hr p=0.8254; sh#1 24hr p=0.0026, 48hr p=0.0004, 72hr p=0.0001; MYCN shEV 24hr p=0.5479, 48hr p=0.7921, 72hr p=0.4004; sh#1 24hr p=0.0499, 48hr p=0.0462,72hr p=0.0363) (**b**) Doxycycline-induced knockdown of RUNX1T1 (72 hrs; 1 µg/mL) in KELLY cells significantly reduced MAX protein levels. Values represent means from 3 independent experiments ± SEM. Two-tailed unpaired t-test shEV p=0.0083, sh#1 p=0.0021. (**c**) Western blots show no significant changes in expression of seven Max binding partners following knockdown of RUNX1T1 with shRNA in KELLY cells. Each experiment has been repeated three times with similar results. (**d**) Co-IP with MYCN antibodies following shRNA knockdown of RUNX1T1 in KELLY cells led to a significant reduction in the ratio MAX:MYCN binding by comparison with control cells (ns p=0.1436, *p=0.0196) and (**e**) significantly decreased binding of MYCN to the promoter of target genes GSPT1, CLNS1A and NAP1L1, as determined by ChIP-qPCR. Two-tailed unpaired t-test GSTP1 ns p=0.3199, **p=0.0054; CLNS1A ** p=0.002, **** p<0.0001; NAP1L1 ns p=0.0715, *** p=0.0007. Values represent means from 3 independent experiments ± SEM.

Supplementary Fig. 4

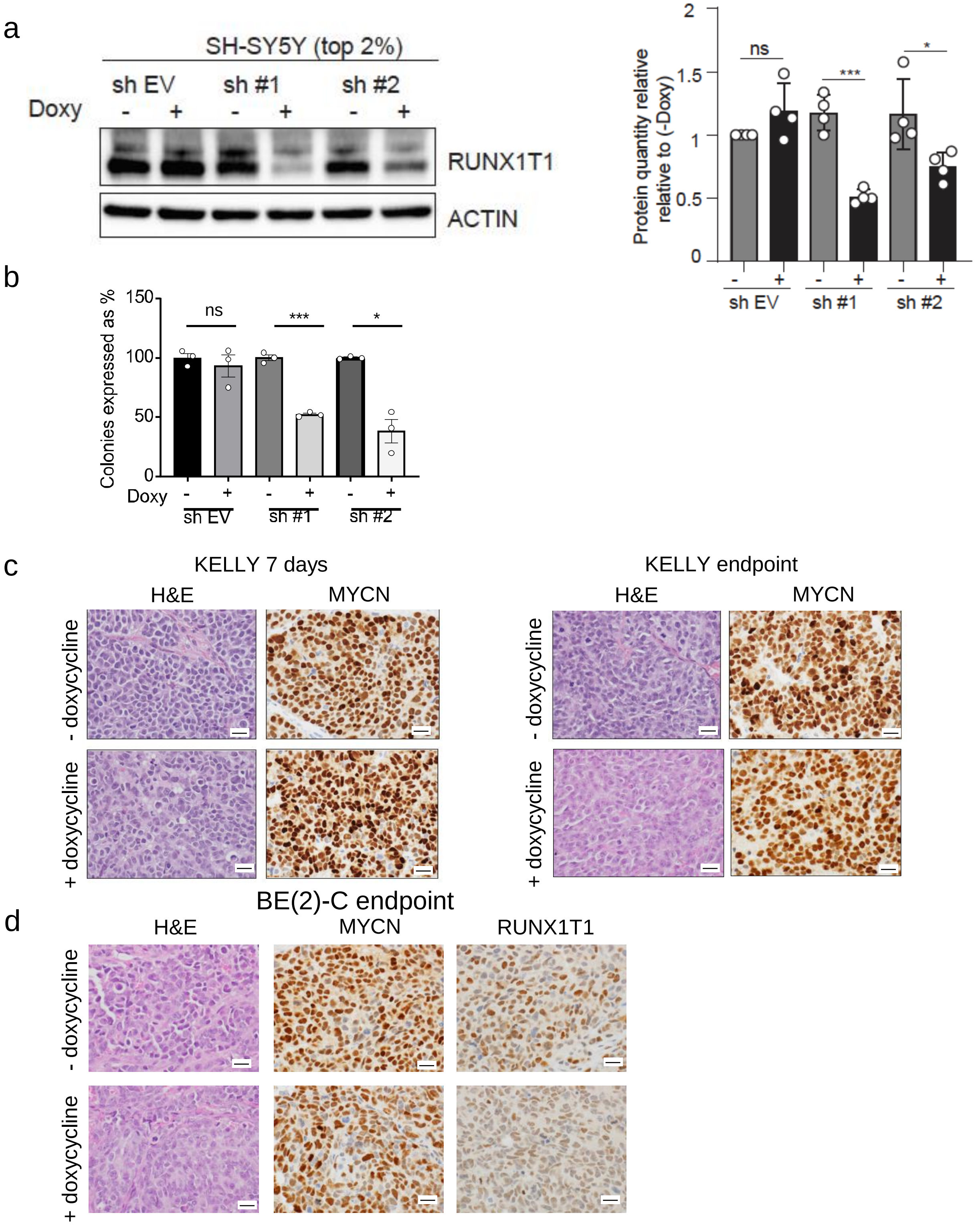

Supplementary Fig. 4: Colony formation and histopathology staining following shRNA knockdown of RUNX1T1. (**a**) RUNX1T1 knock down after 72hrs of doxycycline (1 µg/mL) treatment in SH-SY5Y cells, with two independent shRNA constructs (left panel). Quantitation from four independent experiments (right panel) demonstrated significantly decreased RUNX1T1 with shRNA#1 (*** p=0.0001) and shRNA#2 (* p=0.0327). shEV ns p=0.1298. Two-tailed unpaired t-test. Values represent means from 4 independent experiments ± SD. (**b**) Colony formation following doxycycline-induced knockdown of RUNX1T1 in SH-SY5Y cells, with two independent shRNA constructs. Colonies are represented as a percentage relative to the untreated control from three independent experiments. Quantitation demonstrated a significant decreases in colony number in shRNA#1 (*** p=0.0001) and shRNA#2 (*p=0.0327). shEV ns p=0.1298.Two-tailed unpaired t-test. (**c**) Representative images of xenografted KELLY cells at 7 days post-doxycycline and end-point. Tumors are stained with H&E and MYCN. Scale bar 20µm. (**d**) Representative images of xenografted BE(2)-C tumor cells at end-point post-doxycycline treatment compared to vehicle control. Tissue samples were stained either with H&E or immunohistochemically for MYCN and RUNX1T1. Immunostaining for MYCN protein demonstrated similar strong nuclear staining in both cohorts. Reduced RUNX1T1 staining was observed in tumor cells.

Supplementary Fig. 5

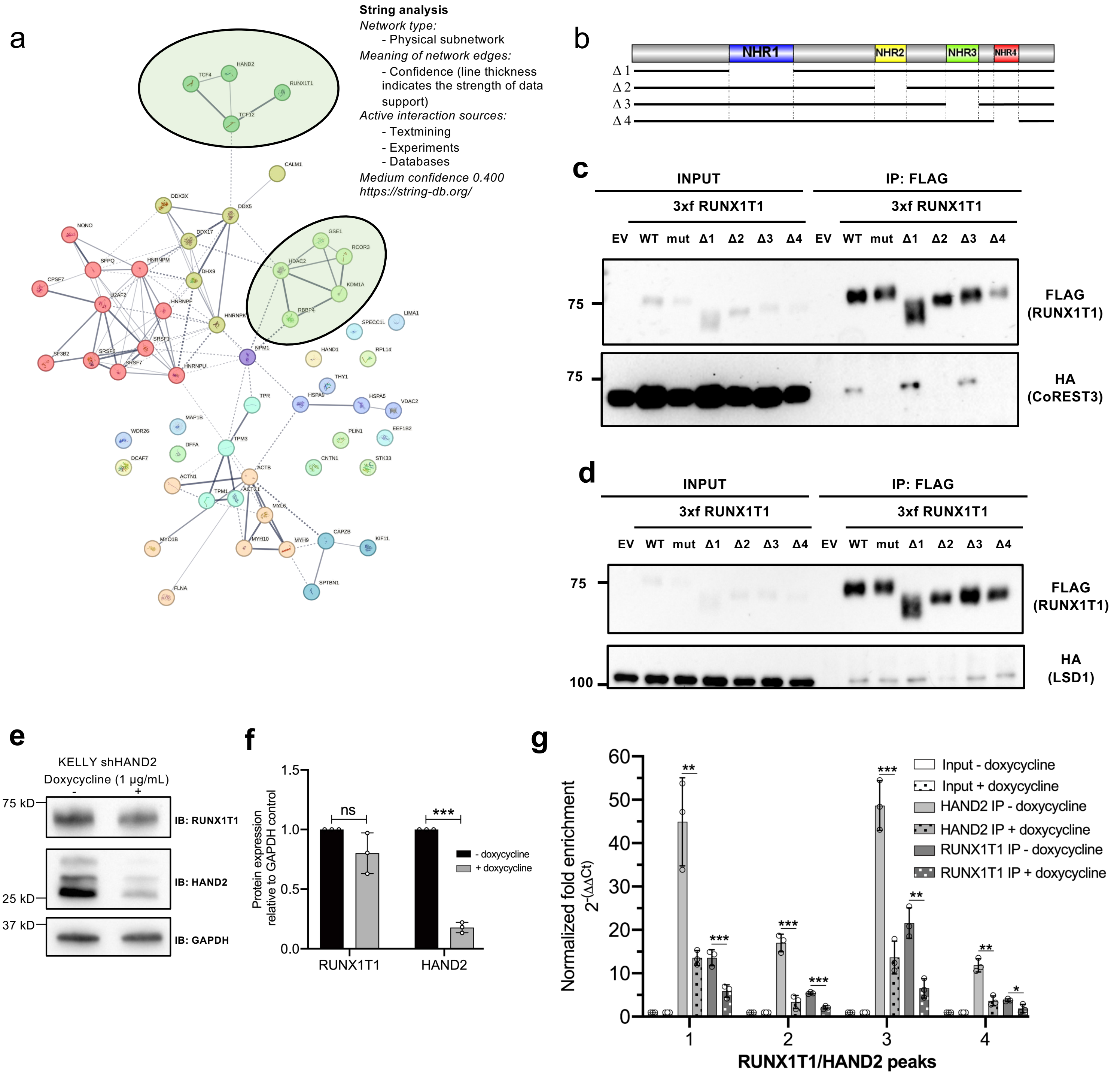

Supplementary Fig. 5: RUNX1T1 forms part of an LCH repressor complex. (**a**) String analysis performed following LC-MS/MS of FLAG-IP samples from BE(2)-C cells transiently (48 hrs) transfected with 3xFLAG-tagged wild type RUNX1T1. Clusters, identified by different colors are putative protein complexes based on published data. DNA binding transcription factors including HAND2 and epigenetic modifiers (HDAC2, KDM1A and RCOR3) are highlighted in the green circles. (**b**) graphical representation of the RUNX1T1 deletion mutants used. (**c**) (**d**) Individual deletion of each of the four RUNX1T1 NHR regions followed by Co-IP with either CoREST3/RCOR3 (**c**) or LSD1 (**d**). n=1 experiment. (**e**) HAND2 Knock-Down with shHAND2 after 72 hrs of doxycycline (1µg/mL) treatment in KELLY cells. (**f**) normalized quantification (relative to GAPDH) from three independent experiments demonstrated significantly decreased HAND2 levels and no significant change in RUNX1T1 following shRNA-mediated HAND2 knockdown. Data are mean ± SD (n = 3 biological replicates; two-tailed unpaired t-test). * p < 0.05; ** p < 0.01; *** p < 0.001. (**g**) RUNX1T1 and HAND2 ChIP-qPCR assays in KELLY cells with HAND2 Tet-inducible KD) cells following doxycyline treatment (1 µg/ml for 48 hrs). Input (white bars), HAND2 IP (light gray bars) and RUNX1T1 IP (dark grey bars) samples were analyzed by q-PCR using specific primers for Peak 1, 2, 3, 4 and ABCA10 TSS as negative control (Supplementary Table 7); dotted bars refers to HAND2 KD condition (+ doxycycline). Data are mean ± SD (n = 3 biological replicates; two-tailed unpaired t-test). * p < 0.05; ** p < 0.01; *** p < 0.001.

Supplementary Fig. 6

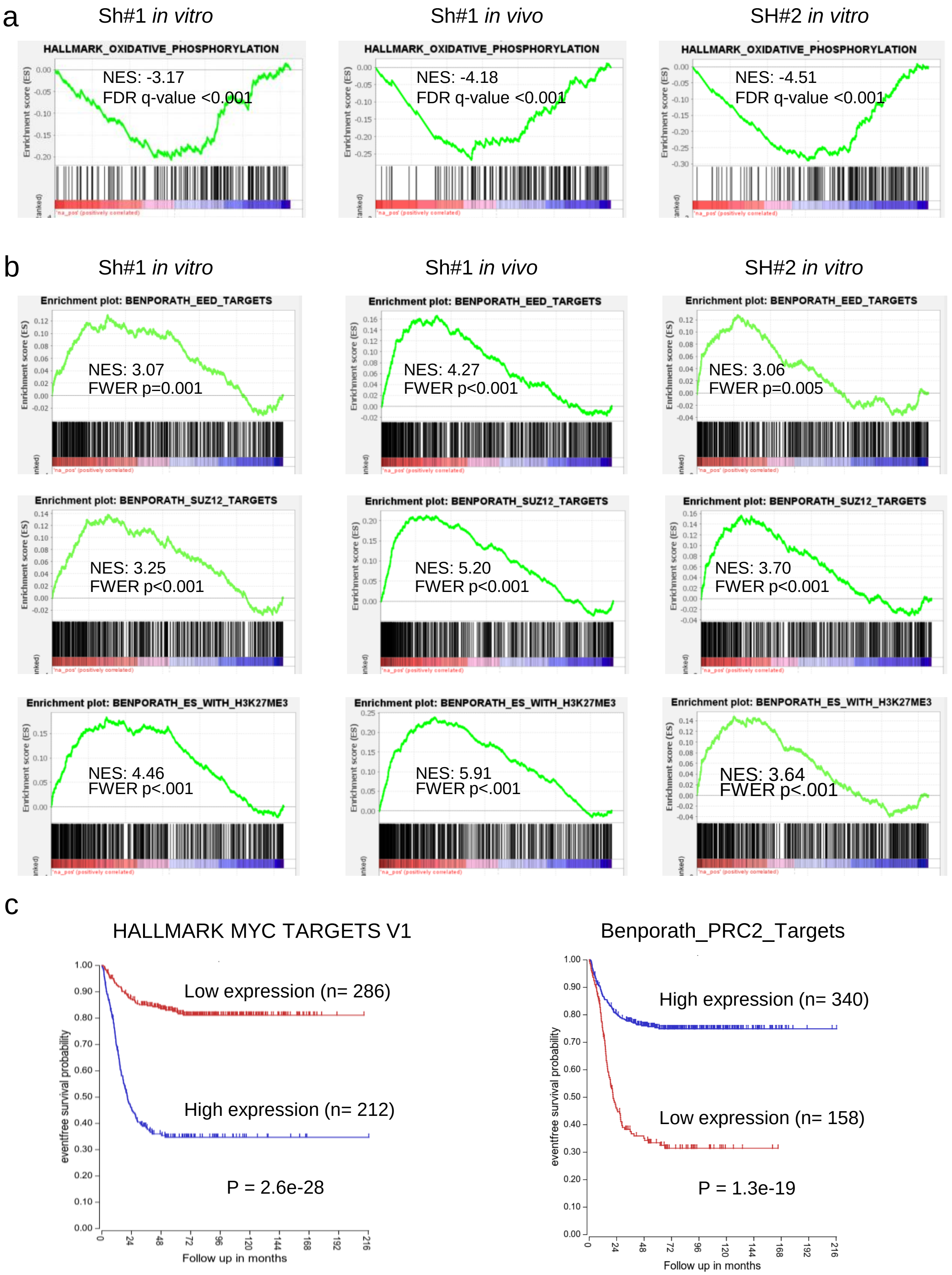

Supplementary Fig. 6: RUNX1T1 depletion downregulates Hallmark oxidative phosphorylation genes and upregulates PRC2 component target genes. (**a**) GSEA demonstrated significant downregulation in expression of Hallmark oxidative phosphorylation genes following *RUNX1T1* shRNA downregulation. (**b**) GSEA showed significant enrichment in expression of PRC2 subunit (*EED, SUZ12* and *H3K27me3*) target genes. (**c**) Kaplan-Meier analysis of GSEA enriched MYC and PRC2 target gene signatures in a publicly available RNAseq data set (SEQC) of tumor samples from the neuroblastoma R2 database showed high expression of MYC and low expression of the PRC2 signatures were highly prognostic of poor outcome.

Supplementary Fig. 7

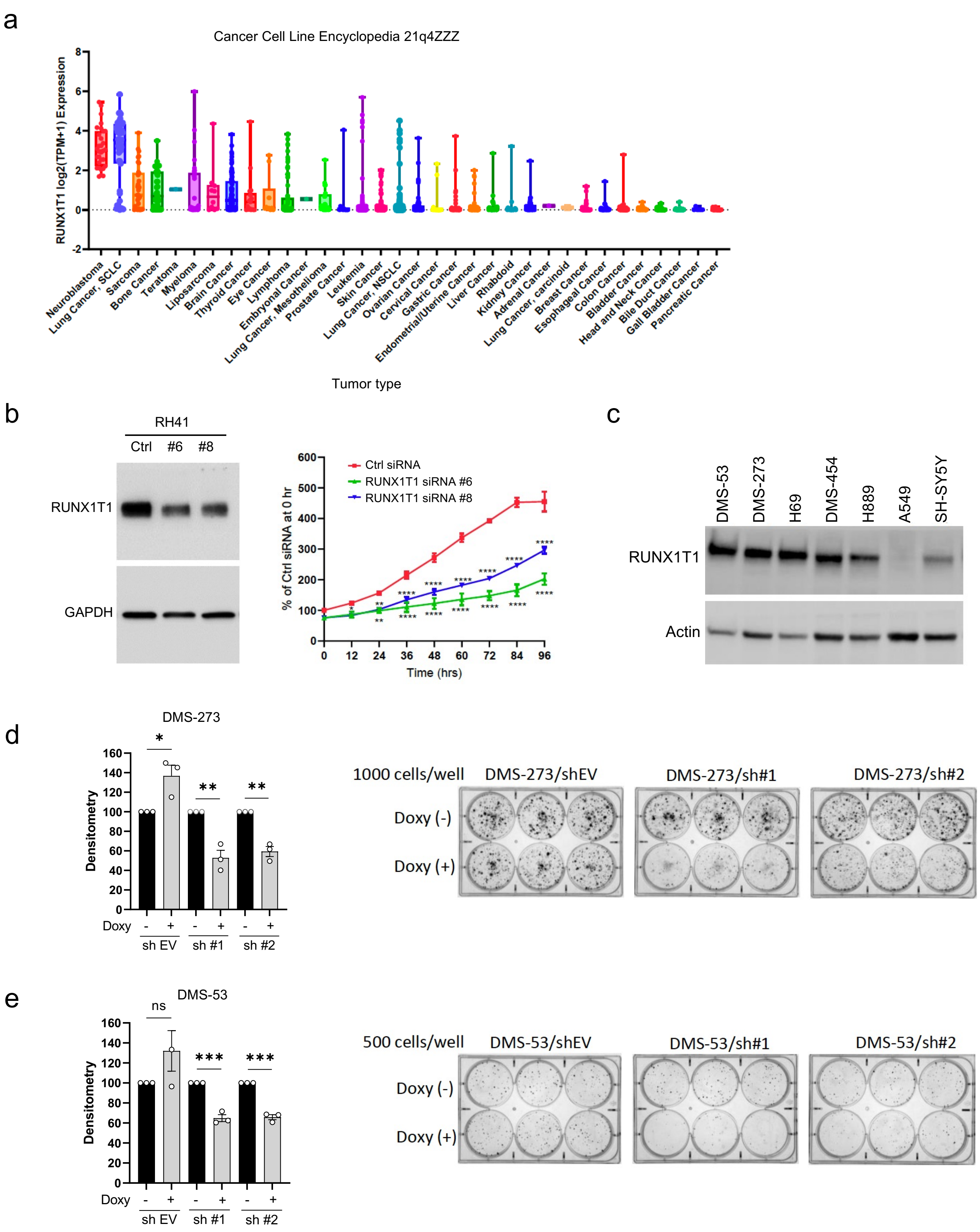

Supplementary Fig. 7: RUNX1T1 loss reduces proliferation in alveolar rhabdomyosarcoma and clonogenic capacity in small cell lung cancer cells. (**a**) Highest level of RUNX1T1 expression in neuroblastoma cell lines followed by SCLC and sarcoma lines in all tumor types in publicly available data from the Cancer Cell Line Encyclopedia (https://depmap.org/portal/download/all/). Box limits indicate 25th and 75th percentile with the central line marking the median value. The whiskers are minimum to maximum, and all points are shown.(**b**) Cell proliferation following knockdown of RUNX1T1 in RH41 rhabdomyosarcoma cells using two independent siRNA sequences. Values represent mean ± SEM from three independent runs. Two-way ANOVA with Dunnett's multiple comparison test 24 hour control versus siRNA #6 **p=0.0039 and versus siRNA #8 p=0.0038, 48-96 hours ****p<0.0001. (**c**) Five SCLC cell lines (DMS- 53, DMS-273, DMS-454, H69, H889) demonstrated high level RUNX1T1 protein expression by comparison with the non- small cell lung cancer (A549) and neuroblastoma (SH-SY5Y) cells. We have run the Western blot of RUNX1T1 on these cell lines two times with similar results. (**d**) (**e**) Protein quantitation (left panels) and colony formation (right panels) following doxycycline-induced knockdown of RUNX1T1 in DMS-273 and DMS-53 SCLC cells, with two independent shRNA constructs. Quantitation of RUNX1T1 protein is expressed as mean ±SD (n=3). Two-tailed unpaired t-test (for DMS-273, shEV *p=0.0284, sh#1 **p=0.0037, sh#2 **p=0.0014; for DMA-53, shEV ns=0.1879, sh#1 ***p=0.0005, sh#2 ***p=0.0002.

Supplementary Table 1 Doxycycline-induced knockdown of RUNX1T1 in KELLY xenografts

Sample ID*	Treatment	Timepoint	Differentiation	Morphology	Ki67 (%)**	Fibrotic stroma***
C1#1	Control	Day 7	Poorly differentiated	Prominent nucleoli	88	++
C1#2	Control	Day 7	Undifferentiated	Prominent nucleoli	70	+
C1#3	Control	Day 7	Poorly differentiated	Prominent nucleoli	87	++
C1#4	Control	Day 7	Poorly differentiated	Prominent nucleoli	77	+++
C2#1	Doxycycline	Day 7	Poorly differentiated	Prominent nucleoli	43	+
C2#2	Doxycycline	Day 7	Poorly differentiated	Prominent nucleoli	47	++
C2#3	Doxycycline	Day 7	Poorly differentiated	Prominent nucleoli	40	++
C2#4	Doxycycline	Day 7	Undifferentiated	Prominent nucleoli	60	++
C1#1	Control	Endpoint	Undifferentiated	Prominent nucleoli	73	+
C1#2	Control	Endpoint	Poorly differentiated	Prominent nucleoli	70	+
C1#3	Control	Endpoint	Undifferentiated	Prominent nucleoli	73	+
C1#4	Control	Endpoint	Poorly differentiated	Prominent nucleoli	75	++
C1#5	Control	Endpoint	Poorly differentiated	Prominent nucleoli	73	+
C2#1	Doxycycline	Endpoint	Poorly differentiated	Prominent nucleoli	63	+
C2#2	Doxycycline	Endpoint	Poorly differentiated	Prominent nucleoli	70	+
C2#3	Doxycycline	Endpoint	Poorly differentiated	Prominent nucleoli	50	+
C2#4	Doxycycline	Endpoint	Poorly differentiated	Prominent nucleoli	67	+
C2#5	Doxycycline	Endpoint	Poorly differentiated	Prominent nucleoli	67	+
C3#1	Control	Endpoint	Poorly differentiated	Prominent nucleoli	70	++
C3#2	Control	Endpoint	Poorly differentiated	Prominent nucleoli	67	+
C3#3	Control	Endpoint	Poorly differentiated	Prominent nucleoli	83	+
C3#4	Control	Endpoint	Undifferentiated	Prominent nucleoli	70	+
C3#5	Control	Endpoint	Undifferentiated	Prominent nucleoli	63	+
C4#1	Doxycycline	Endpoint	Poorly differentiated	Prominent nucleoli	73	+
C4#2	Doxycycline	Endpoint	Poorly differentiated	Prominent nucleoli	67	+
C4#3	Doxycycline	Endpoint	Poorly differentiated	Prominent nucleoli	57	+
C4#4	Doxycycline	Endpoint	Poorly differentiated	Prominent nucleoli	70	+
C4#5	Doxycycline	Endpoint	Poorly differentiated	Prominent nucleoli	67	+

* C1#1, C1#2 etc, refers to cage number and individual mouse within that cage.

** The Ki67 proliferative index (%) was determined by visually estimating the % of viable tumour cells with Ki67 nuclear staining, to the nearest 10%, in tissue microarray cores. Two or three cores were available for assessment for all tumors except C2#4 Day 7 for which only a single core was available. When multiple cores were assessed from the same tumor sample, the values were averaged.

*** Semi-quantitative assessment of extent of fibrocollagenous connective tissue in xenografts where: + <10%; ++ 11-50%; +++ >50%

Supplementary Table 2 Primers and probes used in real-time PCR analysis for mouse genotyping

**Gene**	**Primer/ Probe**	**Sequence**
Th-MYCN		
MYCN	*MYCN* F	5’-CGACCACAAGGCCCTCAGTA
	MYCN R	5’-CAGCCTTGGTGTTGGAGGAG
	Probe	6FAM-CGCTTCTCCACAGTGACCACGTCG TAMRA
**Chr 18***	Chr 18 F	5’- CCACAAAAATATGACTTCCTAAAAGATTT
	Chr 18 R	5’- CATGGGACTTCCTCCTTATATGCT
	Probe	VIC-5’-AACAATTATAACACCATTAGATATG TAMRA
***Runx1t1* Knock-out genotyping**		
Runx1t1	*Runx1t1* F	5’-TGAAGACGCAGTCTAGGCTGACT
	Runx1t1 R	5’-TACTTACATGTCGTTGGCGTAAATG
	Probe	6FAM 5’-CAATGCCACCTCCTC MGB-NFQ†
lacZ	*lacZ* F	5’-CAGAAACAGCACCTCGAACTGA
	lacZ R	5’-GCCATACAGCGCGTTGAAA
	Probe	VIC-5’-CCGCGATATTGCC MGB-NFQ
***Runx1t1* #1590 SNV**		
**#1590 Forward**		5’-GACGTGCAGCGGCTGTAA
**#1590 Reverse**		5’-CCCAGTCTTTATGCTGGCAAA
**WT Probe**		VIC-5’-ACGGCCCGATACT MGB-NFQ†
**Variant Probe**		6FAM-5’-ACGGCCCGACACT MGB-NFQ

* Chr indicates Chromosome

† MGB-NFQ refers to minor groove binder–non fluorescent quencher

Supplementary Table 3 Primers for expression assays

**Primer Name**	**Sequence 5'-3'**
Mm gusb F	TGGCTGGGTGTGGTATGAAC
Mm gusb R	TCCCATTCACCCACACAACT
mRUNX1t1 all F	AGGCGAACTCCAGACAGAAC
mRUNX1t1 all R	AATAGTGCATGGTCGCTTGC
h MYCN qPCR F	CACAAGGCCCTCAGTACCTC
h MYCn qPCR R	ACCACGTCGATTTCTTCCTC
h GUSB Fw	TGGTGCGTAGGGACAAGAAC
h GUSB RV	CCAAGGATTTGGTGTGAGCG
Hs_Runx1t1_All_F	AGTTGCACTAGACGCGCA
Hs_Runx1t1_All_R	TGGCGCTTCACCTCATTGAC

Supplementary Table 4 Primers used to create 5'UTR RUNX1T1 constructs

**Primer Name**	**Sequence 5'-3'**
m5UTR_ iso1 F H3	AAAAGCTTCCTGATGCACGTTGGCTCCTCTCC
m5UTR_ iso1 R H3	AA**AAGCTT**tctccagcaagcgctgtgctaatc
m5UTR_ iso2 F H3	AAAAGCTTGAGGGCGGGCGAGGCGGAC
m5UTR_ iso2 R H3	AA**AAGCTT**CCGCCGCGGGCTCCCAGC
m5UTR_ iso3 F H3	AAAAGCTTCTGGAACTGGGGCAGGAGGAAGAG
m5UTR_ iso3 R H3	AA**AAGCTT**cccggaatccagcgtgtaacacac

Supplementary Table 5 Sequence of oligos used to create RUNX1T1 shRNA constructs

**Primer Name**	**Sequence 5'-3'**
shRNA#l *RUNX1T1* Ambion Forward	**TCCCGGTTCCTTACTACCCTGCA**TTCAAGAGA*TGCAGGGT AGTAAGGAACC*TTTTTC
shRNA#l *RUNX1T1* Ambion Reverse	TCGAGAAAAA*GGTTCCTTACTACCCTGCA*TCTCTTGAA**TG CAGGGTAGTAAGGAACC**
shRNA#2 *RUNX1T1* Forward	TCCC**AAGCAAGCGACCATGCACTA**TTTCAAGAGA*ATAGTG CATGGTCGCTTGCTT*TTTTTC
shRNA#2 *RUNX1T1* Reverse	TCGAGAAA*AAAAGCAAGCGACCATGCACTAT*TCTCTTGA A**ATAGTGCATGGTCGCTTGCTT**
shRNA *HAND2* Forward	TCCC*TCAAGAAGACCGACGTG*AAAGCTCGAG**CTTTCACGT CG GTCTTCTTGA**TTTTTTC
shRNA *HAND2* Reverse	TCGAGAAAAAA*TCAAGAAGACCGACGTGAAA*GCTCGAG**C TTTCACGTCGGTCTTCTTGA**

Bases in Bold: sense or passenger (same as transcript)
Bases in Italics: antisense or guide (real siRNA)

Supplementary Table 6 Primers used to create mutant RUNX1T1 constructs

**Primer Name**	**Sequence 5'-3'**
Forward containing *Not1* sequence (in bold)	AA**GCGGCCGC**TCTGTCAAAAGAAACACTTGG
Reverse containing *BamH1* sequence (in bold)	AA**GGATCC**CTAGCGAGGCGTCGTCTCTATG
Forward *Runx1t1* Mutant (YH) (mutant base in bold)	**C**ACTGTGGCTCTTTTTGCCAGC
Reverse *Runx1t1* Mutant (YH)	TCGGGCCGTGTTACAGCCGCTG

Supplementary Table 7 Primers for Max ChIP-qPCR assays

Primer Name	Sequence 5'-3'
GSPT1 Fw	TTCGCCTCGGTAGTTCTCTG
GSPT1 Rev	TGTCCCTGAACTTCGACTCC
CLNS1A Fw	ACCCCGGAAGAACAACTTAG
CLNS1A Rev	GCCGCTTTTCCTAGAATGTC
NAP1L1 Fw	AGTTTGAAACCCAGGGACAG
NAP1L1 Rev	CCTCGGTCTGTGCTTACTTTG
ABCA10 Fw	AGCAACATCACCAACCTTATATTTCCC
ABCA10 Rev	TTAGTCAGTAAACACTCACTCAGTAAAGC
RUNX1T1 peak1 Fw	ACTTGCACAAACCAGAGCTG
RUNX1T1 peak1 Rv	AGGACTTCCGCCTTGTAATGAC
RUNX1T1 peak2 Fw	AGGGCGTCATCCTCTTCTTG
RUNX1T1 peak2 Rv	TGGCCAGGCTTTTATTGTGC
RUNX1T1 peak3 Fw	AATCCCAACCCCCGCATATG
RUNX1T1 peak3 Rv	AAGGCTGCATGGAGAAAAGC
RUNX1T1 peak4 Fw	CACACACACACATACACACCAC
RUNX1T1 peak4 Rv	CTGCCCACGAGAGCTACAAG

Supplementary Table 8 Antibodies used

Protein	Catalogue #	Company	Host	Application	Dilution
MAX (S-20)	#4739S	Cell Signalling	Rabbit monoclonal	WB	1:500
MXD1	19547-1-AP	Proteintech	Rabbit, polyclonal	WB	1:500
MXD2	A12098	Abclonal	Rabbit, polyclonal	WB	1:2000
MXD3	249041	United States Biological/Assay Matrix Pty Ltd.	Mouse, polyclonal	WB	1:500
MXD4	ab220495	Abcam	Rabbit, polyclonal	WB	1:1000
MGA (MXD5) MGA6A4H5	sc-81105	Santa Cruz/Bio-Strategy PTY Limited	Mouse monoclonal	WB	1:250
MNT(MXD6)	MBS9606130	MyBioSource, Inc/Jomar Life Research	Rabbit, polyclonal	WB	1:1000
MLX(MXD7) (F-12)	sc-393086	Santa Cruz	Mouse monoclonal	WB	1:300
RUNX1T1	15494-1-AP	Proteintech	Rabbit, polyclonal	WB	1:1000
RUNX1T1	15494-1-AP	Proteintech	Rabbit, polyclonal	IHC	1:400
MYCN (B8.4.B)	sc-53993	Santa Cruz	Mouse monoclonal	WB	1:1000
MYCN (NCM II 100)	ab16898	Abcam	Mouse monoclonal	IHC	1:200 (TMA)
MYCN	10159-2-AP	Proteintech	Rabbit, polyclonal	IHC	1:1000 (mouse tissues)
B-III Tubulin	802001	Biolegend	Rabbit, polyclonal	IF	1:1000
GAPDH (G-9)	sc-365062	Santa Cruz	Mouse monoclonal	WB	1:5000
ACTIN	A2066	Sigma-Aldrich	Rabbit, polyclonal	WB	1:5000
FLAG (M2)	F3165	Sigma-Aldrich	Mouse monoclonal	Co-IP	1.5ug
MYCN (B8.4.B)	sc-53993	Santa Cruz	Mouse monoclonal	Co-IP	2ug
IgG	sc-2025	Santa Cruz	Mouse monoclonal	Co-IP	2ug
HAND2	EPR19451	Abcam	Rabbit Monoclonal	Co-IP	1:1000
Anti-HA	ab9110	Abcam	Rabbit Polyclonal	Co-IP	1:3000

Source data for Supp Figure 1 can be found with Figure 1

Source data for Supp Figure 2a: Hyperplastic ganglia of 1590 cohort can be found with Figure 2a and 2b

Source Data

Supp Figure 2b
Th-MYCN Runx1t1 WT and YH RUNX1T1 and ACTIN

Run

11/01/2018 Densitometry used 4 brains from different animals

Runx1t1 +/+ Runx1t1 (+/+ Y534H)

RUNX1T1 Ladder 1 2 3 4 1 2 3 4 Ladder

150kD
100kD
75kD
50kD

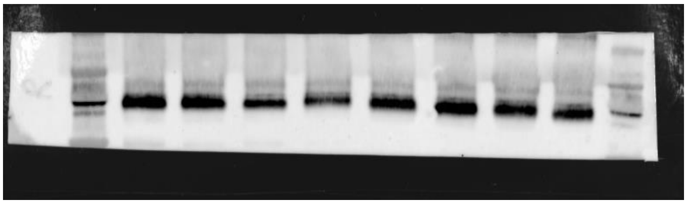

Runx1t1 +/+ Runx1t1 (+/+ Y534H)

ACTIN Ladder 1 2 3 4 1 2 3 4 Ladder

50kD
37kD
25kD

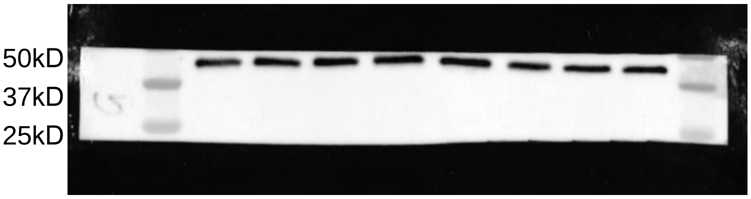

Supp Figure 3a
Kelly time course 0-72 hours, Control shEV and RUNX1T1 sh#1

Run 5/10/2017

RUNX1T1

Control RUNX1T1 sh#1

Ladder 0 24 48 72 0 24 48 72 Ladder

100kD
75kD

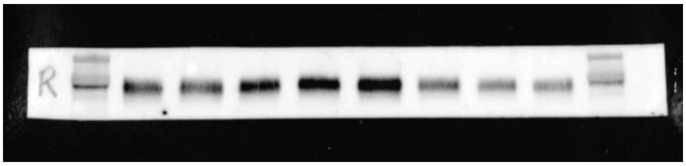

MYCN

Control RUNX1T1 sh#1

Ladder 0 24 48 72 0 24 48 72 Ladder

100kD
75kD

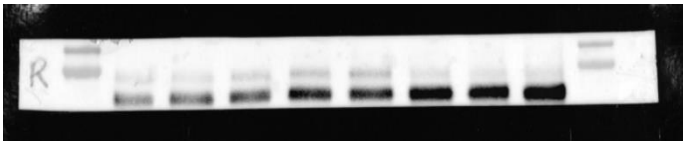

GAPDH

Control RUNX1T1 sh#1

Ladder 0 24 48 72 0 24 48 72 Ladder

50kD
37kD
25kD

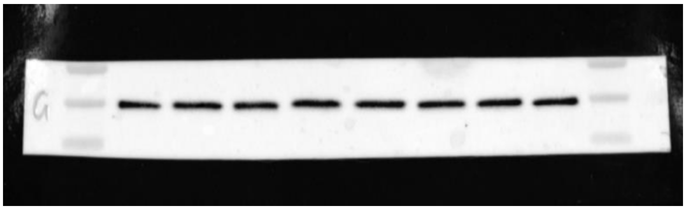

Supp Figure 3a continued
Kelly time course 0-72 hours, Control shEV and RUNX1T1 sh#1 4/08/2023

Note: Samples in gel#1 and gel#2 are from two independent experiments
Run 2 and 3

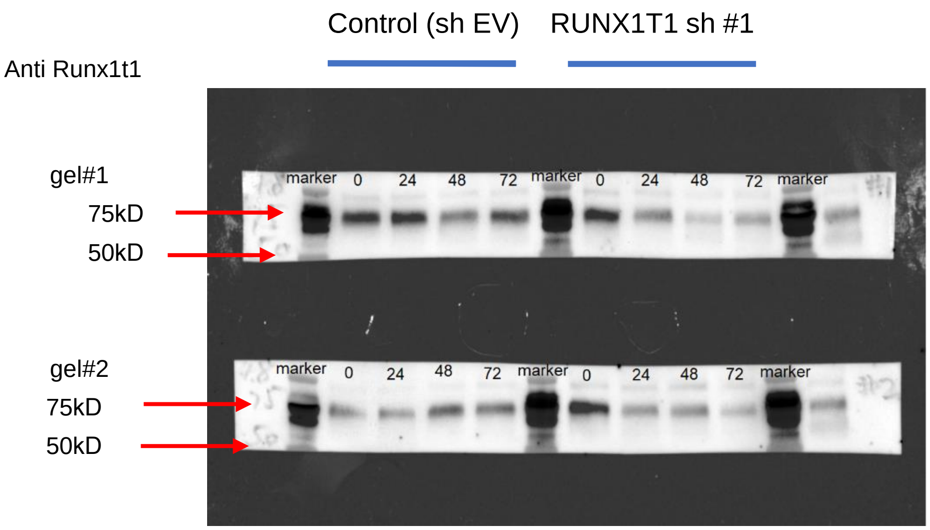

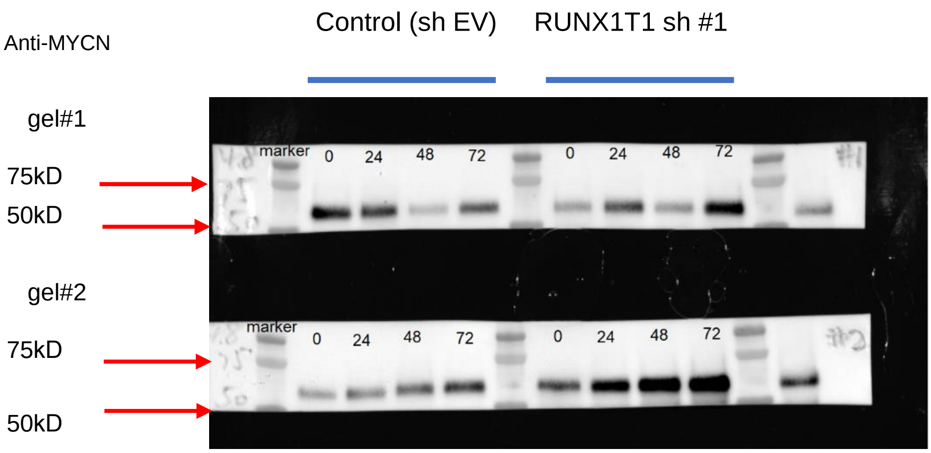

Supp Figure 3b
KELLY +/- Dox Control, sh1, Hela MAX and GAPDH

22/11/2021

gel#1

Kelly cells

SH-SY5Y

Anti-Max

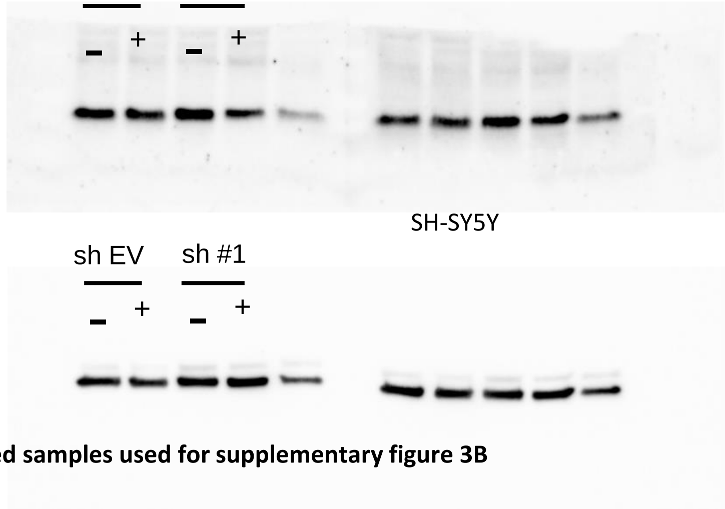

Anti-GAPDH

SH-SY5Y

Labelled samples used for supplementary figure 3B

22/11/2021

gel#1

Merged with protein marker

37kD

Anti-Max25kD

20kD

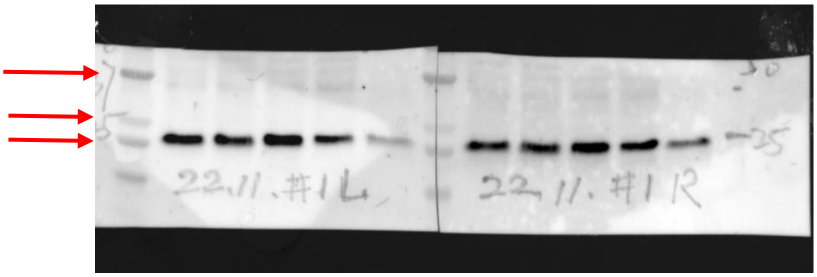

37kD

Anti-GAPDH

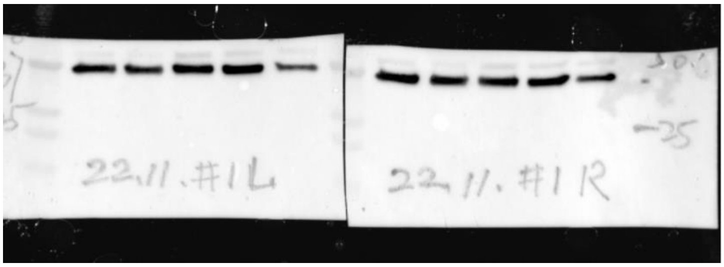

Supp Figure 3b continued
KELLY +/- Dox Control, sh1, Hela MAX and GAPDH

7/12/2021
gel#1

Kelly cells Hela

Anti-Max

sh EV sh #1

– + – +

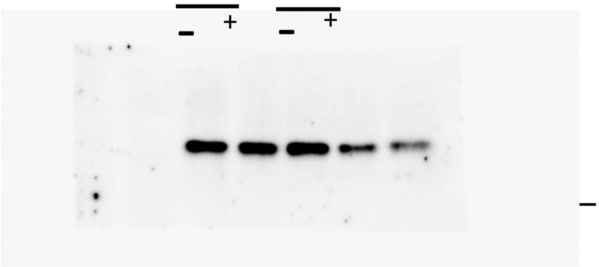

sh EV sh #1

– + – + Hela

Anti-GAPDH

gel#1

gel#2

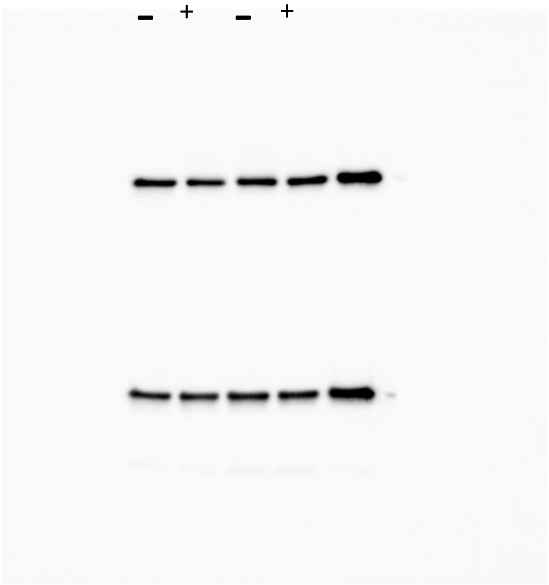

Note: gel#1 used for supplementary figure 3B

7/12/2021
gel#1

Anti-Max
37kD
25kD
20kD

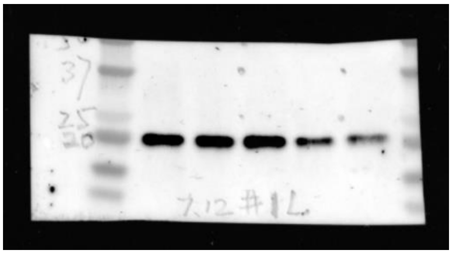

Anti-GAPDH
gel#1

37kD

gel#2

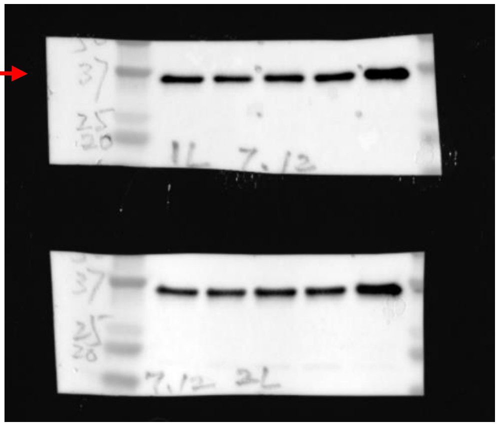

Merged with protein marker

Supp Figure 3c
MAX binding partners

RUNX1T1

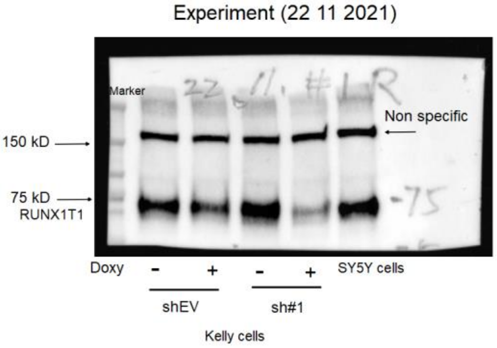

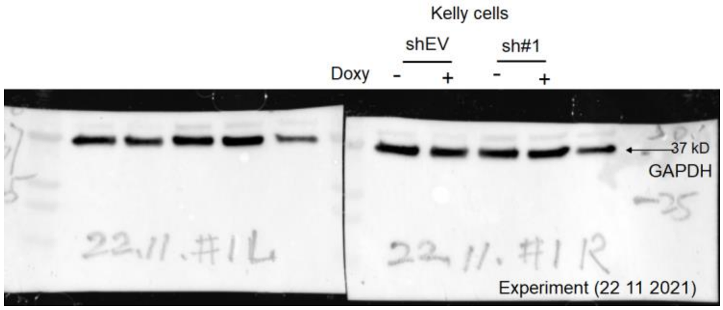

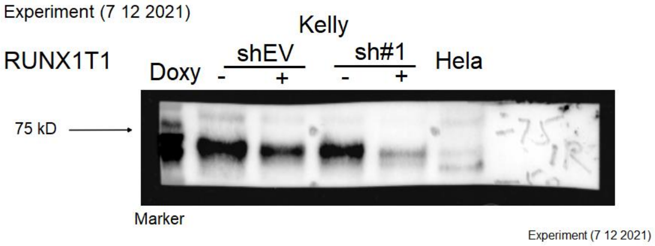

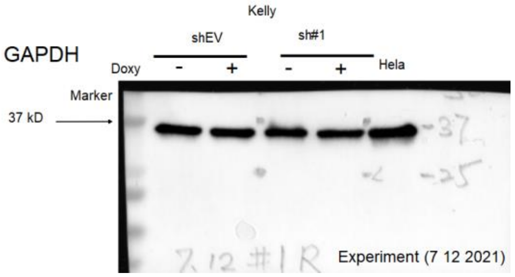

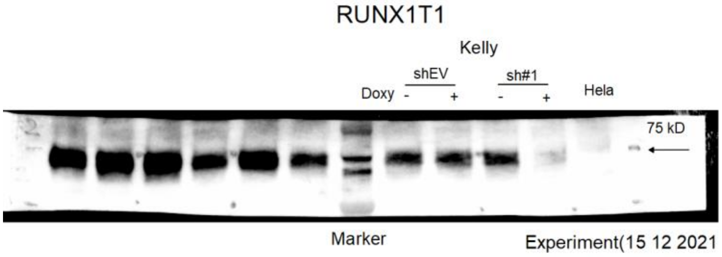

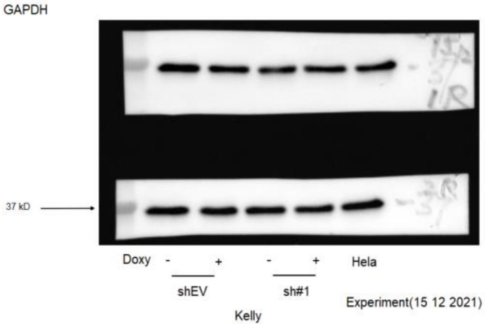

Supp Figure 3c continued
MAX binding partners

MXD1

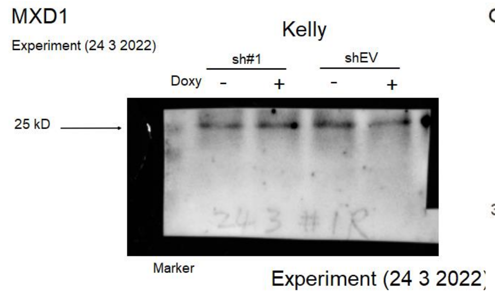

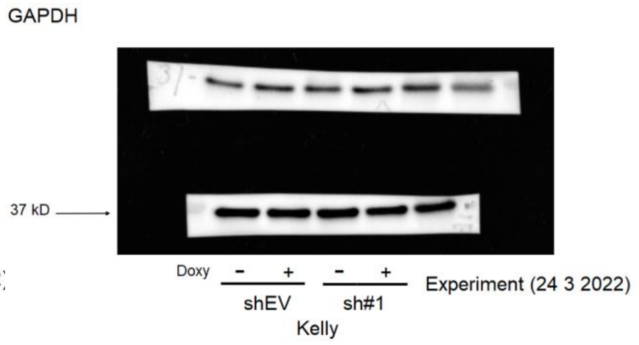

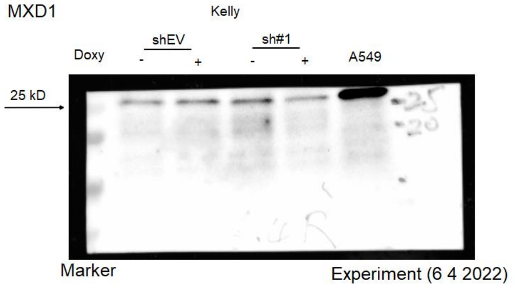

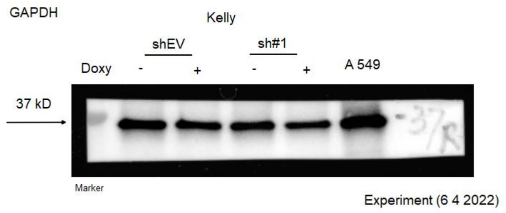

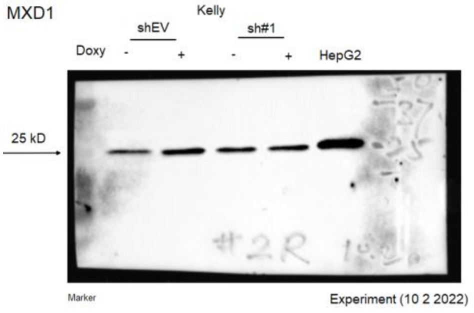

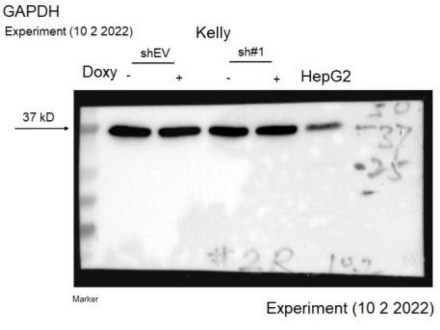

Supp Figure 3c continued
MAX binding partners

MXD2

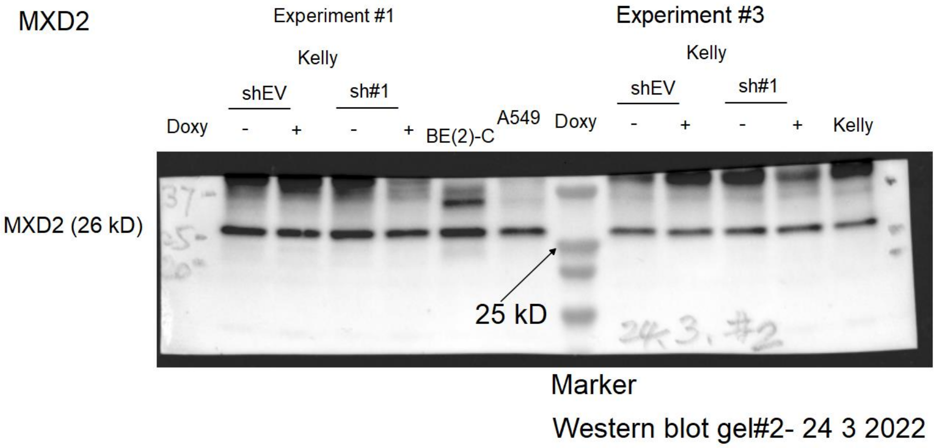

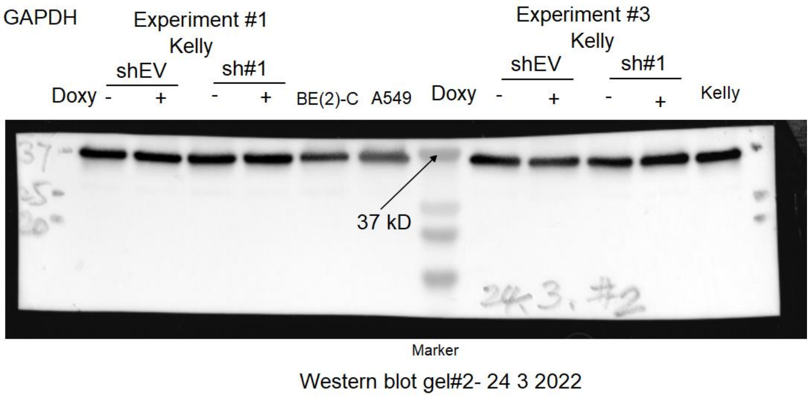

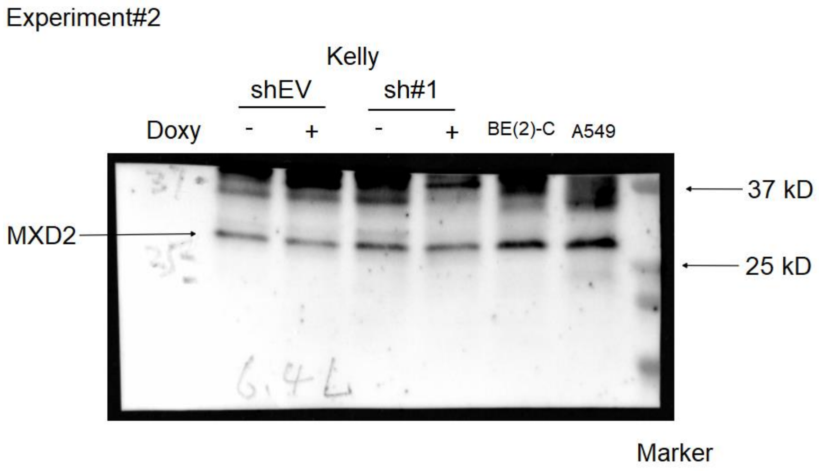

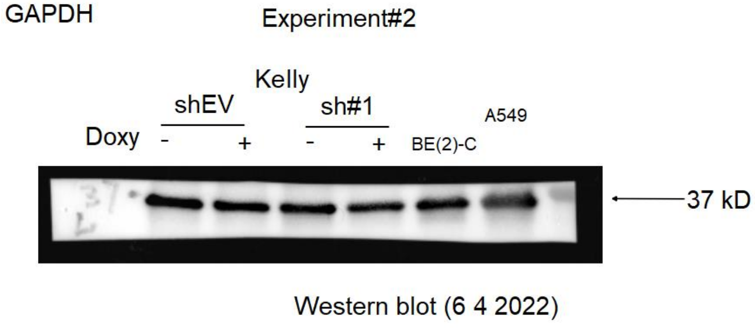

Supp Figure 3c continued
MAX binding partners

MXD3

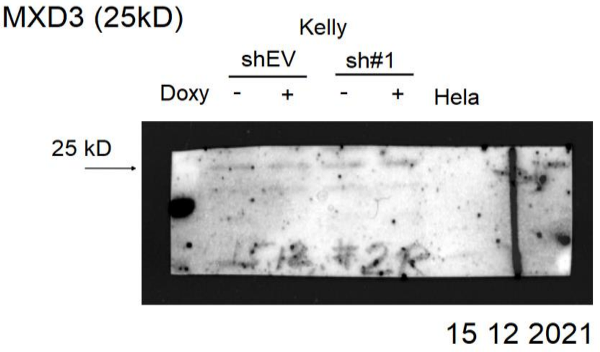

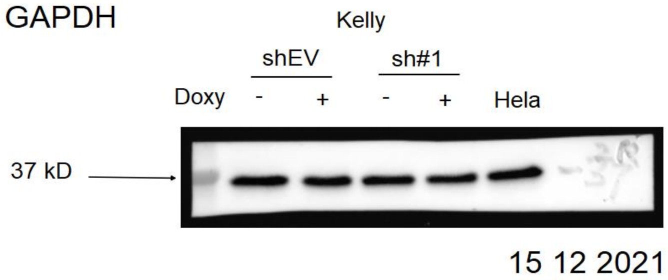

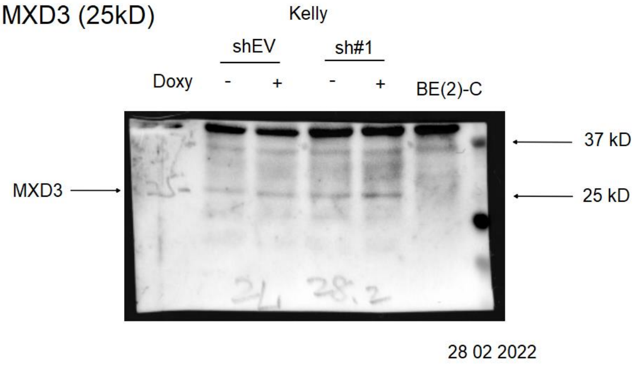

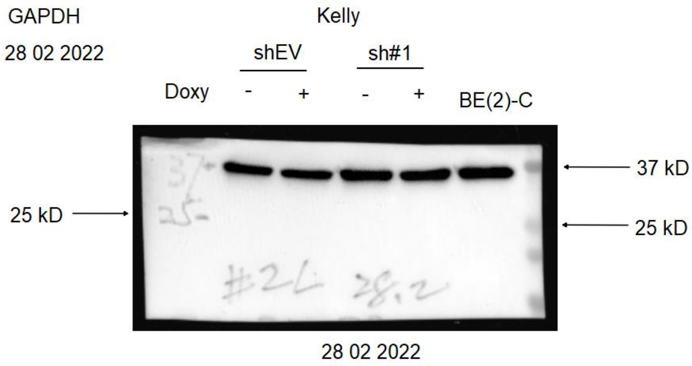

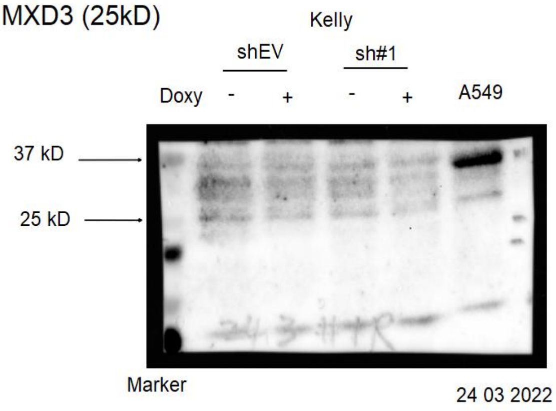

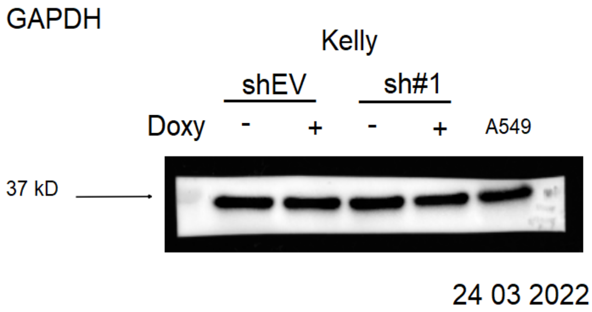

Supp Figure 3c continued
MAX binding partners

MXD4

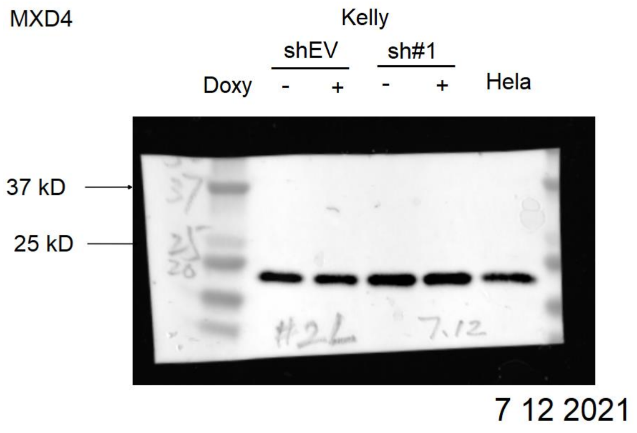

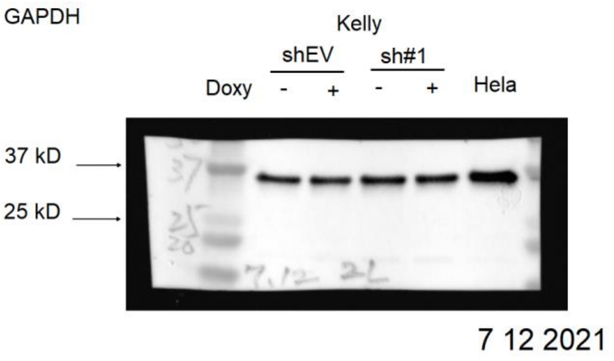

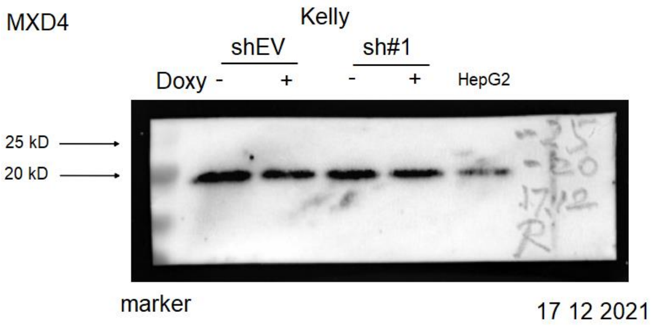

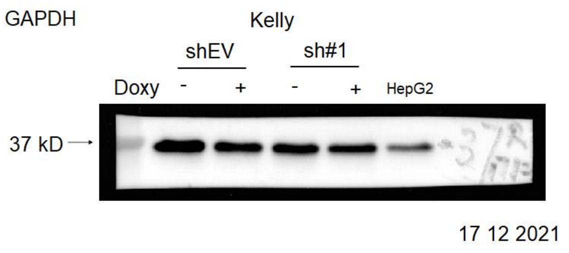

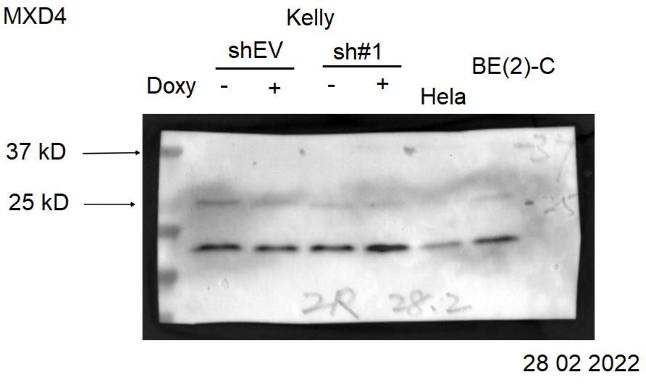

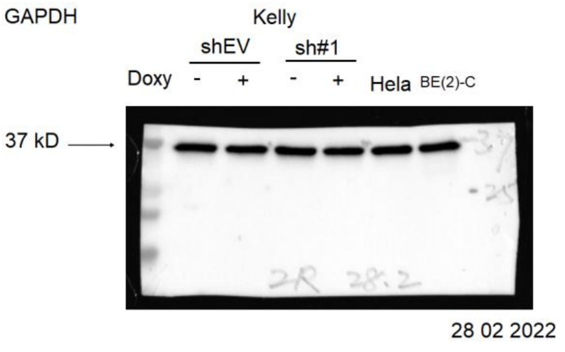

Supp Figure 3c continued
MAX binding partners

MXD5

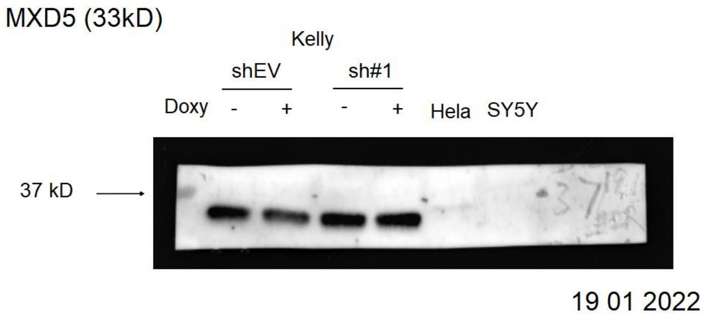

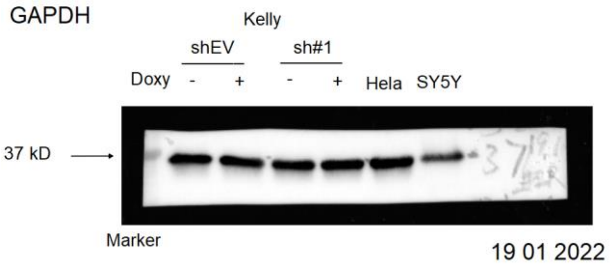

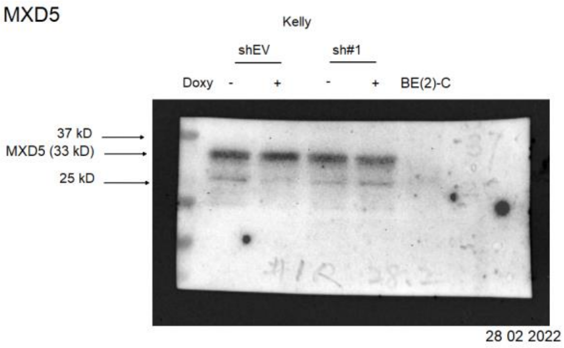

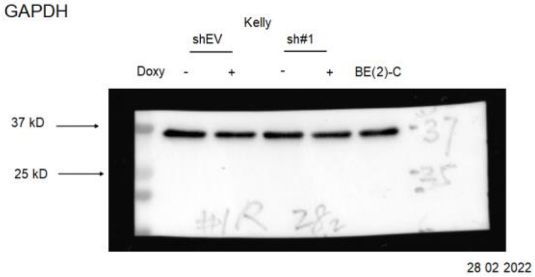

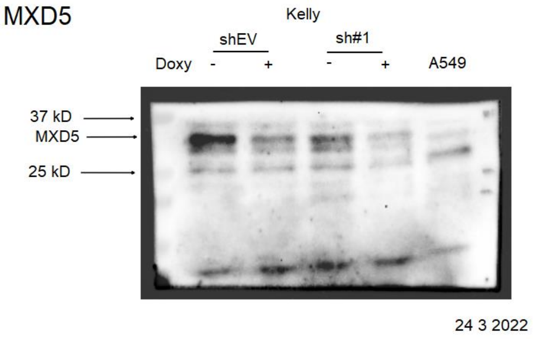

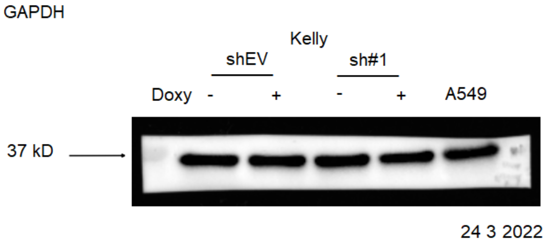

Supp Figure 3c continued
MAX binding partners

MXD6

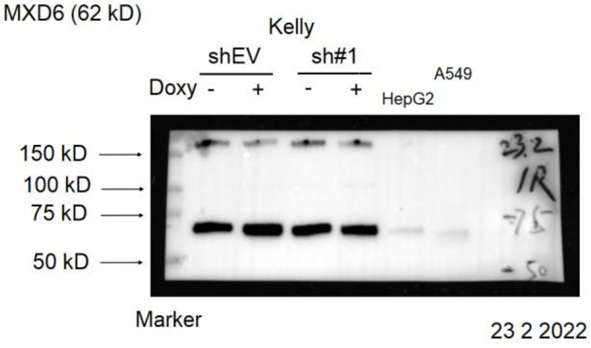

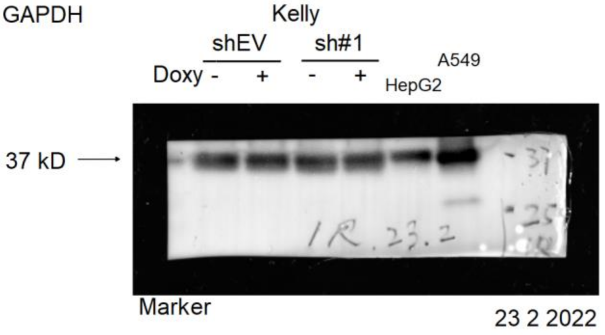

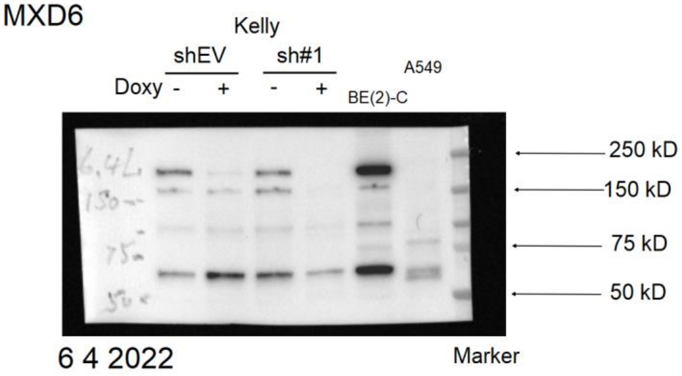

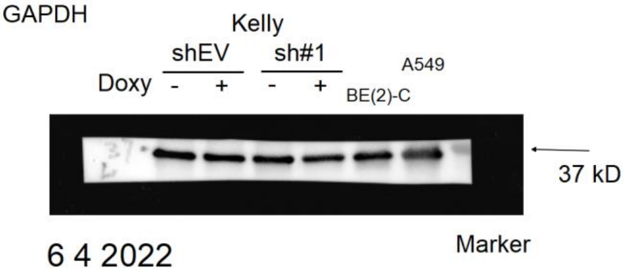

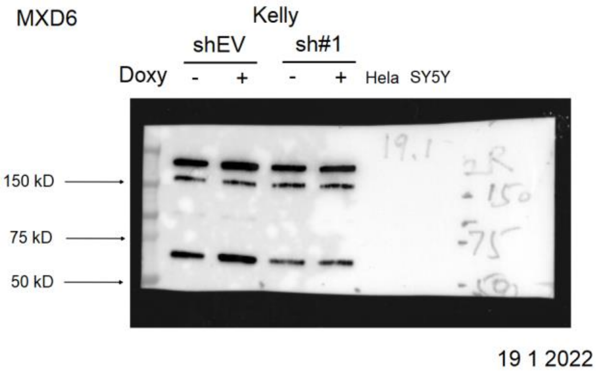

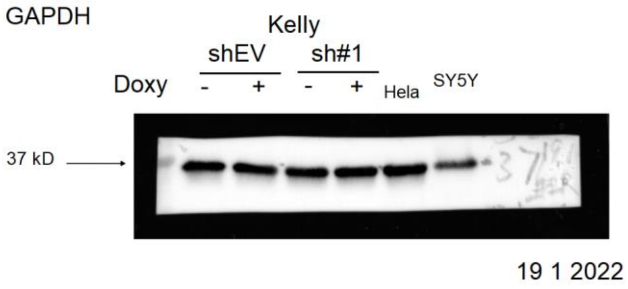

Supp Figure 3c continued
MAX binding partners

MXD7

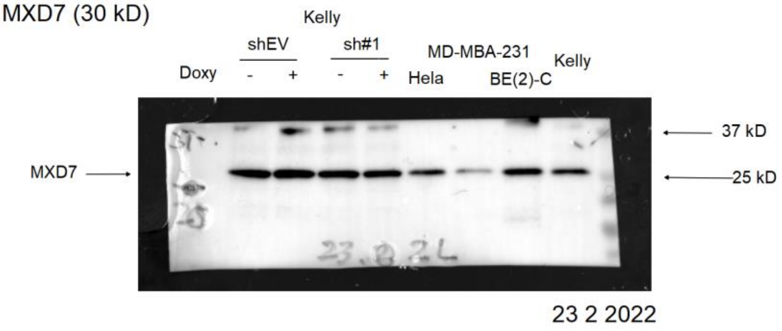

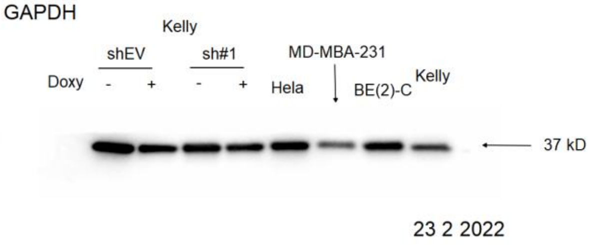

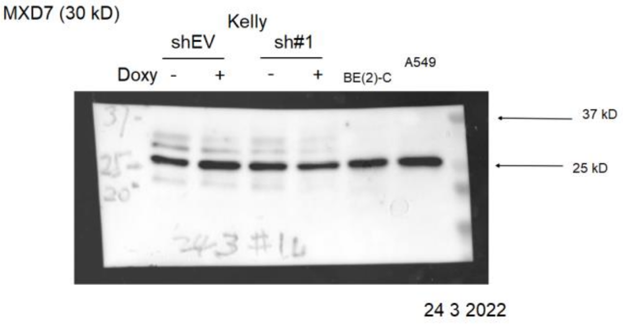

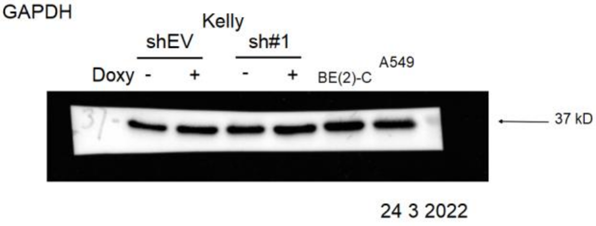

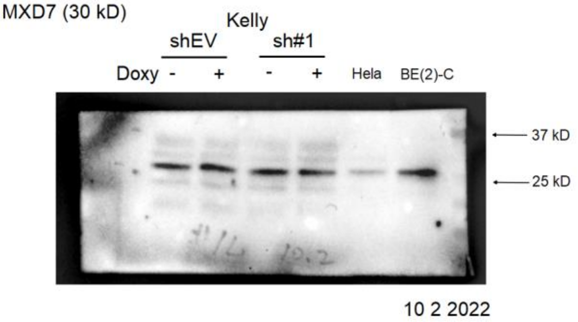

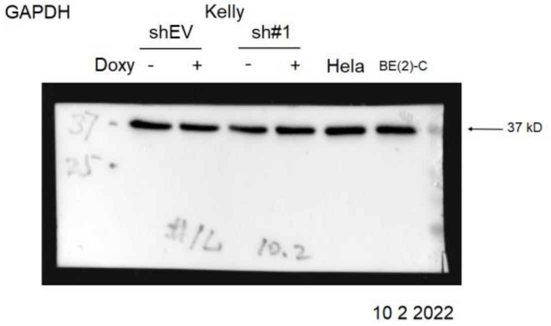

Supp Figure 3d continued
KELLY +/- Dox Control, sh1, Hela MAX and GAPDH

8/03/2022

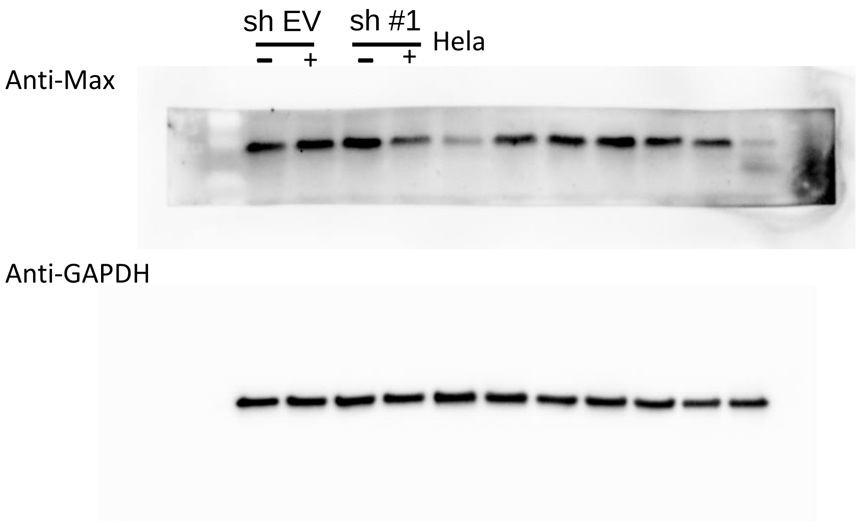

Labeled samples used for supplementary figure 3B

8/03/2022

Merged with protein marker

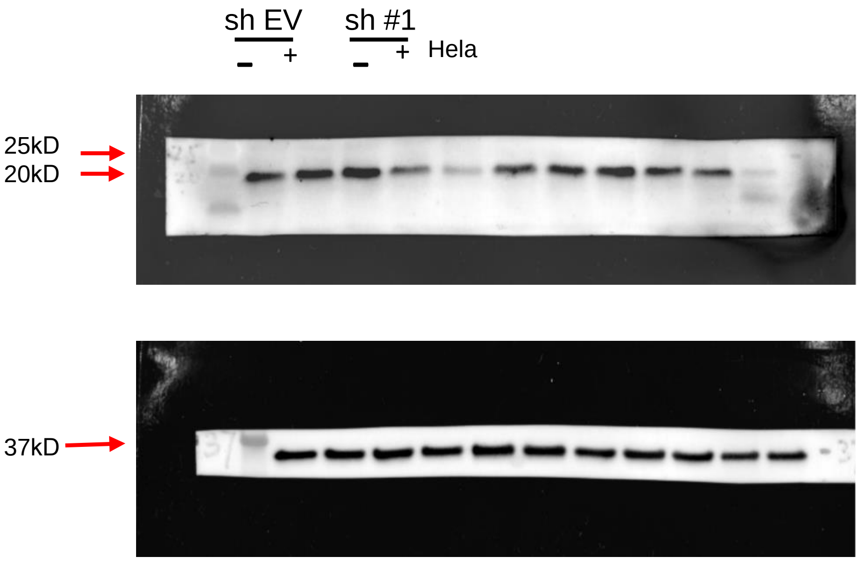

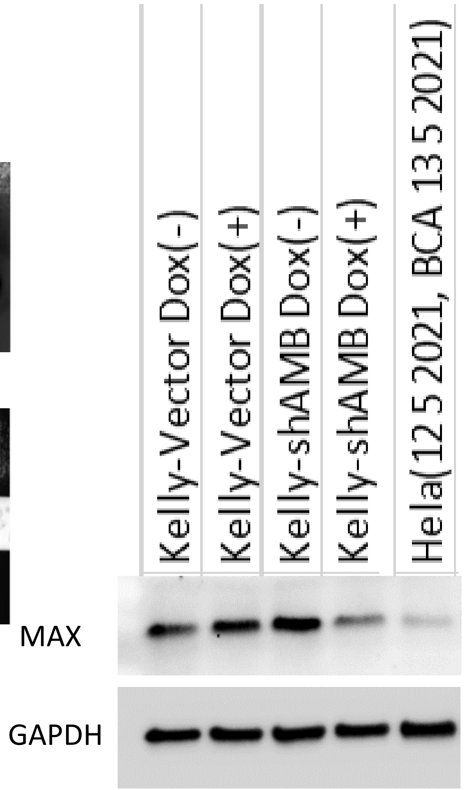

Run#3
(Western-8 3 2022)

Supp Figure 3d
Co-IP MYCN and MAX

IP:MYCN

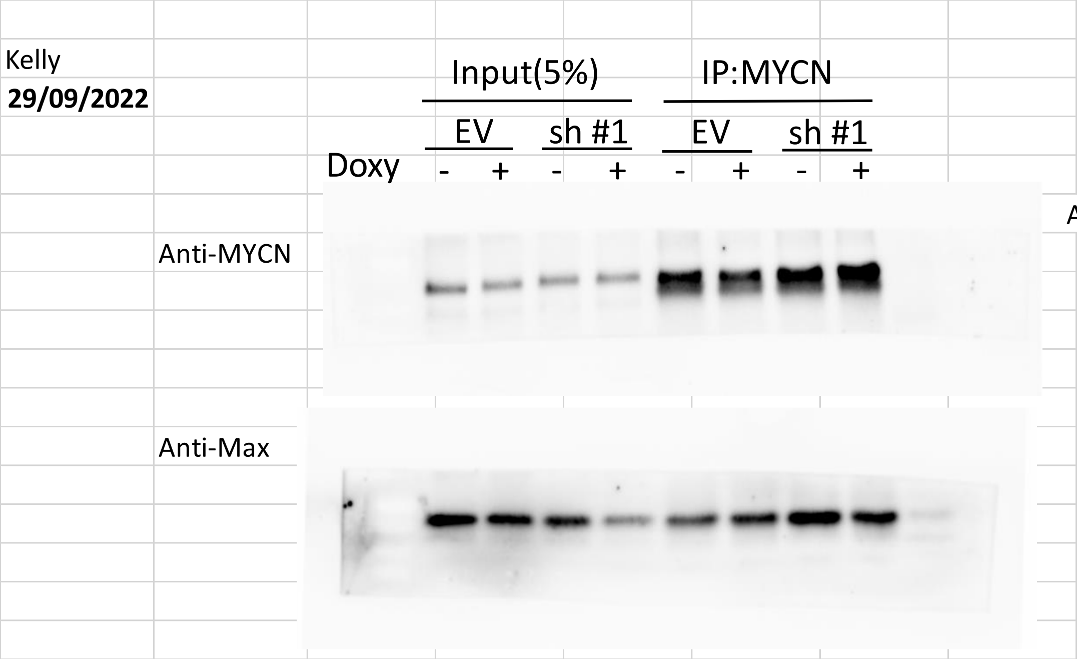

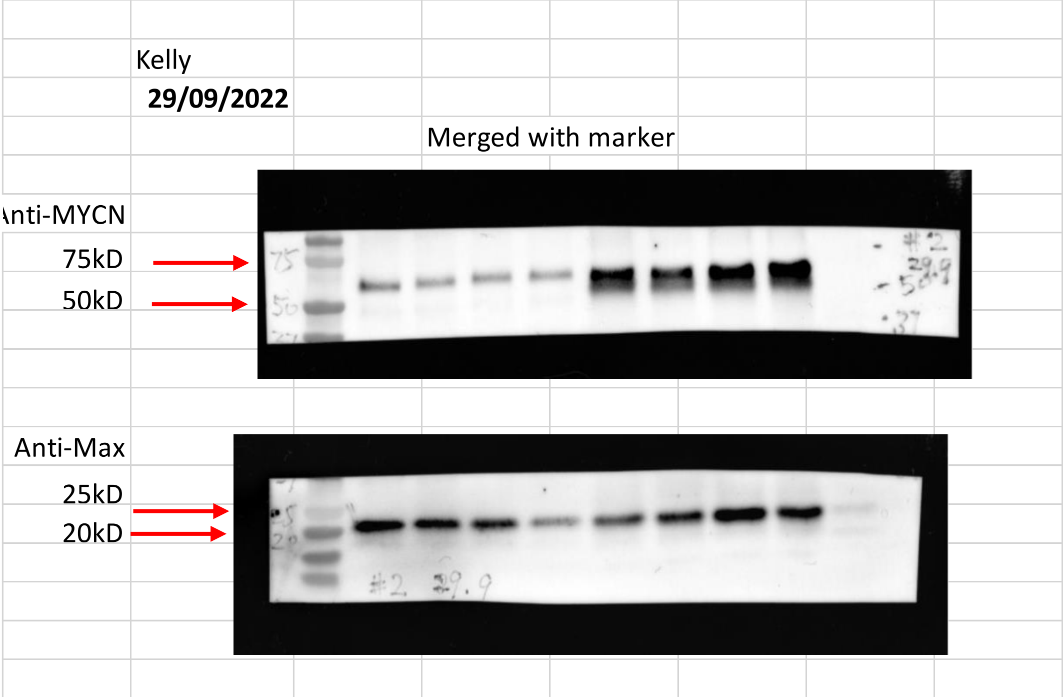

Supp Figure 3d continued
Co-IP MYCN and MAX

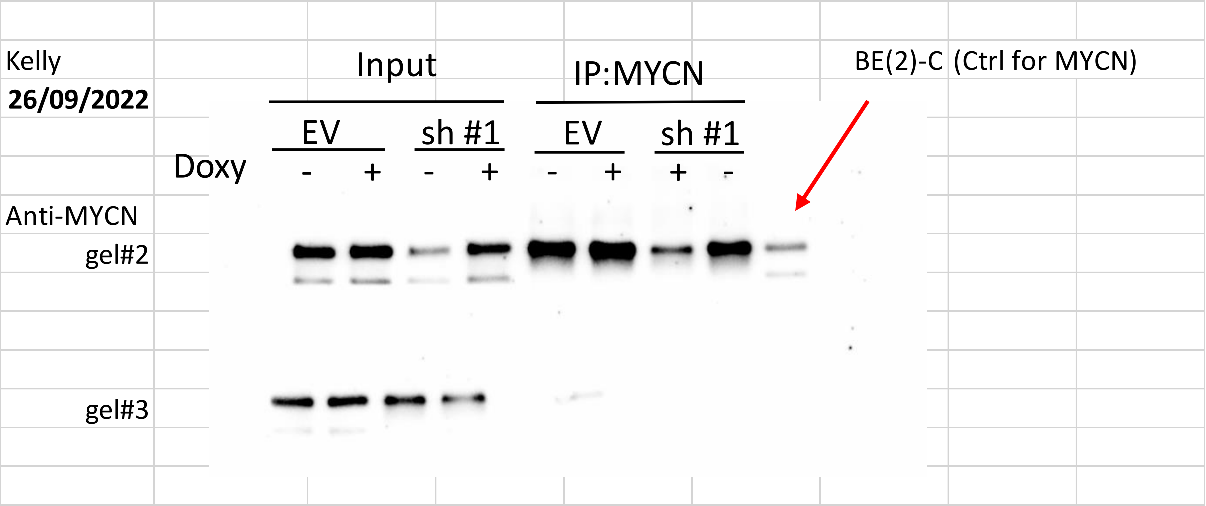

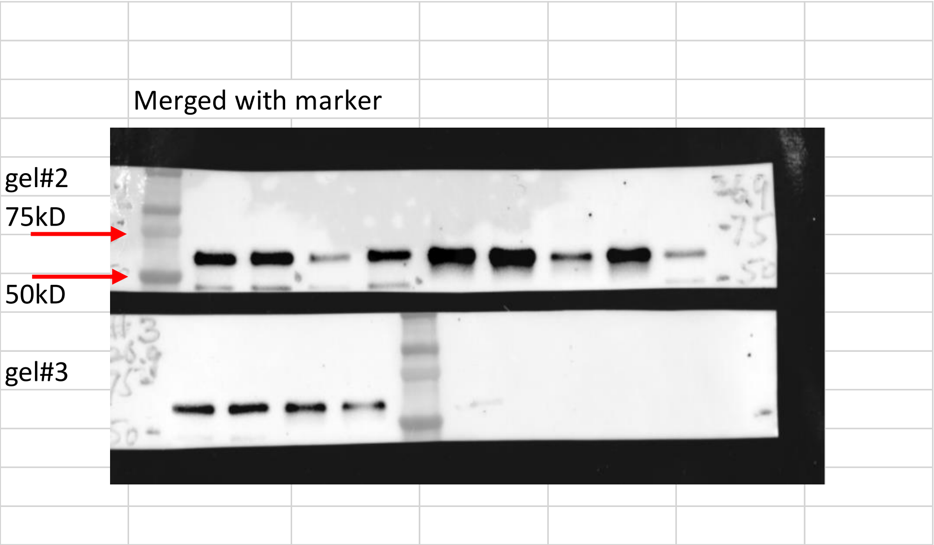

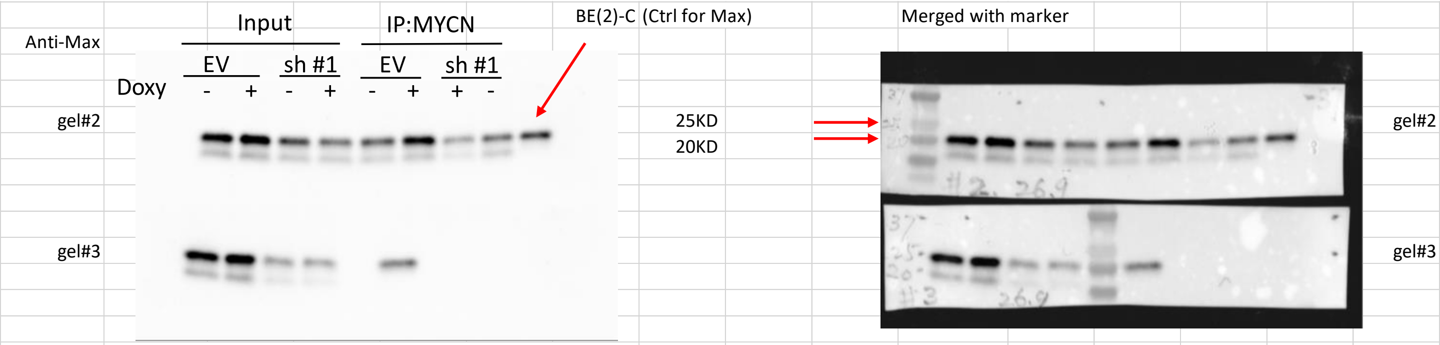

Note: gel#2 was used for Supplementary Fig. #C bar chart

Supp Figure 3d continued
Co-IP MYCN and MAX

Kelly

14/03/2022

Anti-MYCN

gel#1

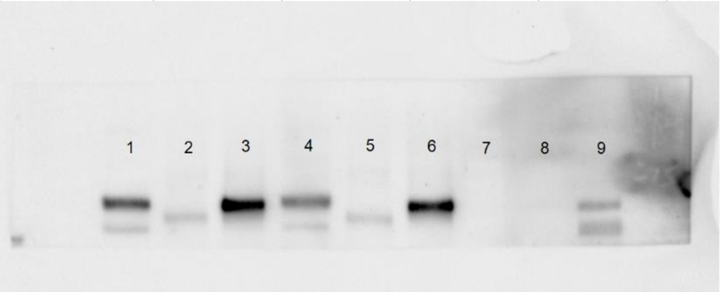

gel#2

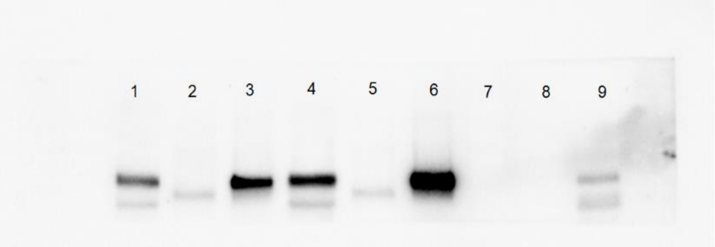

Anti-Max

gel#1

gel#2

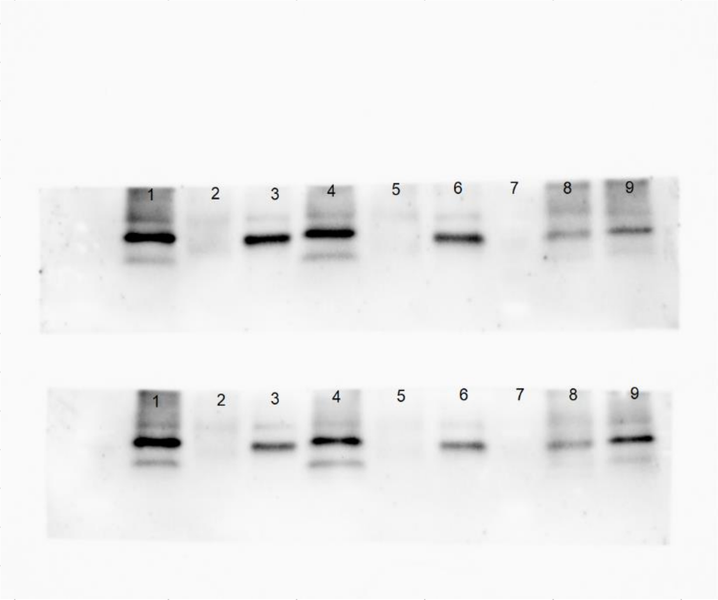

Supp Figure 3d
Co-IP MYCN and MAX (Kelly 14/03/2022)

Merged with marker

gel#1

75kD

50kD

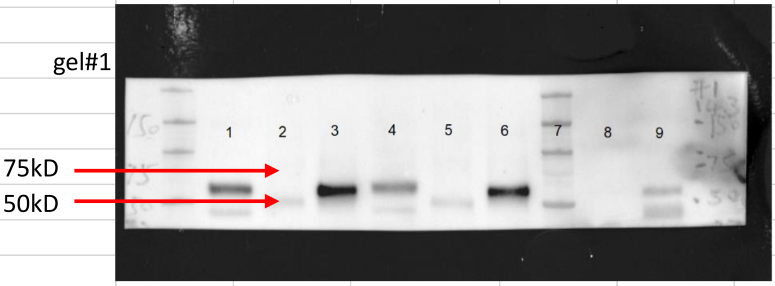

gel#2

75kD

50kD

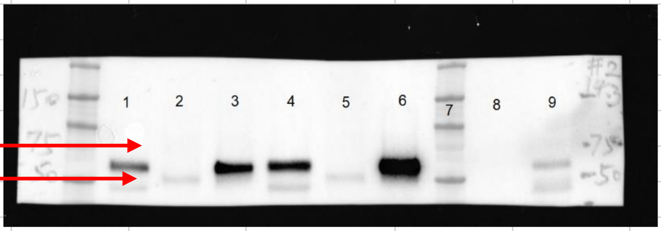

Lane (left to right)

1	Input
2	IgG Ctrl
3	N-Myc IP
4	Input
5	IgG Ctrl
6	N-Myc IP
7	Protein marker
8	Hela (negative Ctrl for MYCN)
9	Kelly (positive Ctrl for MYCN)

Merged with marker

Anti-Max

gel#1

25kD

20kD

gel#2

25kD

20kD

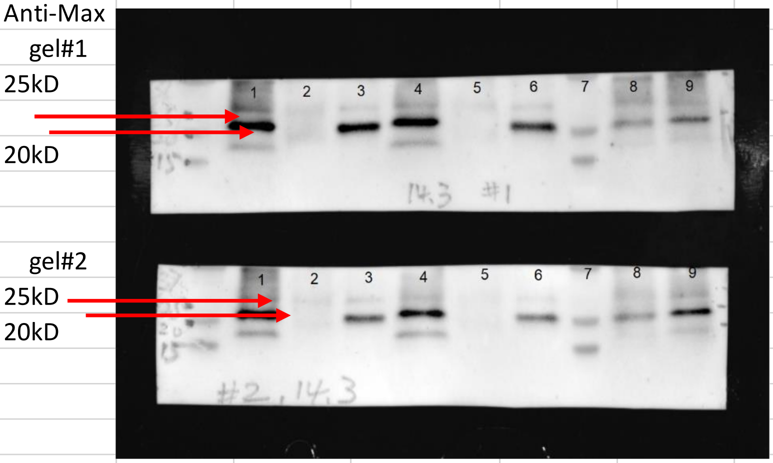

Lane (left to right)

1	Input
2	IgG Ctrl
3	N-Myc IP
4	Input
5	IgG Ctrl
6	N-Myc IP
7	Protein marker
8	Hela (Ctrl for Max)
9	Kelly

Supp Figure 3e
MYCN promotor targets

From File named (shAMBPos - F1HUTGPos) - (shAMBNeg - F1HUTGNeg)_tpm_spia_kegg_go

genes	entrez_id	logFC	logCPM	LR	PValue	FDR	FC	
GSPT1	2935	-1.2101	8.2221	121.8671	2.47E-28	2.05E-26	-2.314	
CLNS1A	1207	-0.9988	7.6921	90.2715	2.08E-21	9.71E-20	-1.998	
NAP1L1	4673	-1.0298	10.2684	89.8286	2.60E-21	1.21E-19	-2.042	
APEX1	328	-0.3399	8.1752	10.3217	0.001	0.004	-1.266	
TFAP4	7023	-0.0923	3.7110	0.1463	0.702	0.782	-1.066	
MAX	4149	-0.695	5.580	37.143	1.10E-09	1.30E-08	-1.619	
RUNX1T1	862	0.189	5.259	2.460	0.117	0.195	1.140	
NTRK1	4914	2.6852	2.9938	50.4777	1.21E-12	2.20E-11	6.432	Confirmed by Western in Kelly

From File named shAMBPos - shAMBNeg_tpm_spia_kegg_go

genes	entrez_id	logFC	logCPM	LR	PValue	FDR	FC	
GSPT1	2935	-1.282	8.222	266.418	0.000	6.82E-58	-2.431	
CLNS1A	1207	-1.076	7.692	205.693	0.000	6.94E-45	-2.109	
NAP1L1	4673	-1.104	10.268	202.726	0.000	2.97E-44	-2.150	
APEX1	328	-0.225	8.175	9.056	0.003	0.005	-1.169	
TFAP4	Not found							
MAX	4149	-0.623	5.580	58.945	1.62E-14	1.34E-13	-1.540	
RUNX1T1	862	0.026	5.259	0.095	0.758031911	0.797	1.018	
NTRK1	4914	3.163	2.994	127.779	1.25E-29	3.17E-28	8.958	Confirmed by Western in Kelly

Gene expression in vector control of shRNA Seq, from file "F1HUTGPos - F1HUTGNeg_tpm"

genes	entrez_id	logFC	logCPM	LR	PValue	FDR	FC
**GSPT1**	2935	-0.071651399	8.222058511	0.863104586	0.352871351	0.7565982	-1.050918943
**CLNS1A**	1207	-0.07744534	7.692095616	1.093632294	0.295667809	0.7565982	-1.055147977
**NAP1L1**	4673	-0.074435657	10.26844727	0.943921451	0.331271381	0.7565982	-1.052949071
**APEX1**	328	0.114728462	8.175193999	2.352291941	0.12509896	0.7565982	1.082771232
**TFAP4**	7023	0.27792397	3.711041207	2.653809674	0.10330206	0.7565982	1.212448922
**MYCN**	4613	0.061924261	11.59586714	0.568396003	0.450897007	0.778420951	1.043857125
**MAX**	4149	0.0720874	5.580076539	0.808114975	0.368678146	0.7565982	1.051236592

Supp Figure 4a
SY5Y: shEV, Sh#1, sh#2 RUNX1T1 and ACTIN

27/05/2019 SH-SY5Y cells

sh EV sh #1 sh #2

Doxy – + – + – +

Anti-Runx1t1

100 KD

75 KD

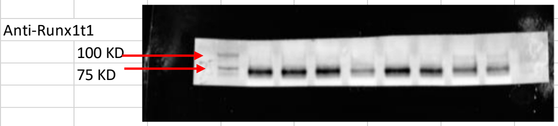

sh EV sh #1 sh #2

Doxy – + – + – +

Anti-Actin

50 KD

37 KD

25 KD

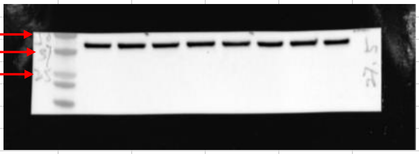

Blot merged with protein marker

3/10/2019 SH-SY5Y cells

sh EV sh #1 sh #2

Doxy – + – + – +

Anti-Runx1t1

75 KD

50 KD

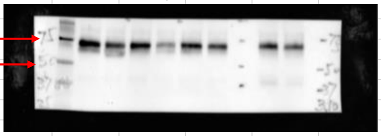

Anti-Actin

50 KD

37 KD

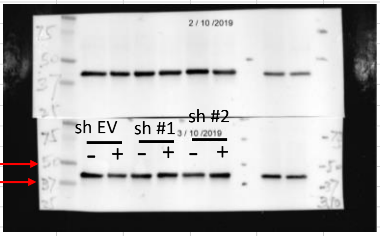

Blot of 2/10/2-19 (not used for figure)

Blot of 3/10/2019

Blot merged with protein marker

17/02/2020
gel#2

SH-SY5Y cells

sh EV sh #1 sh #2

– + – + – +

Anti-Runx1t1

75 KD

50 KD

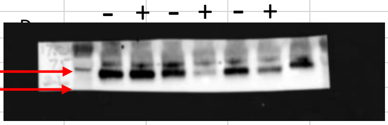

sh EV sh #1 sh #2

– + – + – +

Anti-Actin

50 KD

37 KD

25 KD

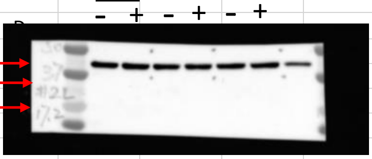

Blot merged with protein marker

11/03/2020
gel#1

SH-SY5Y cells

sh EV sh #1 sh #2

– + – + – +

Anti-Runx1t1

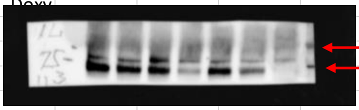

100 KD

75 KD

Anti-Actin

sh EV sh #1 sh #2

– + – + – +

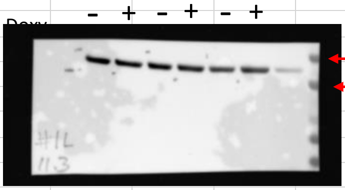

50 KD

37 KD

Blot merged with protein marker

Supp Figure 4b
SY5Y shRNA colonies

SY5Y FH1UTG RUN 1

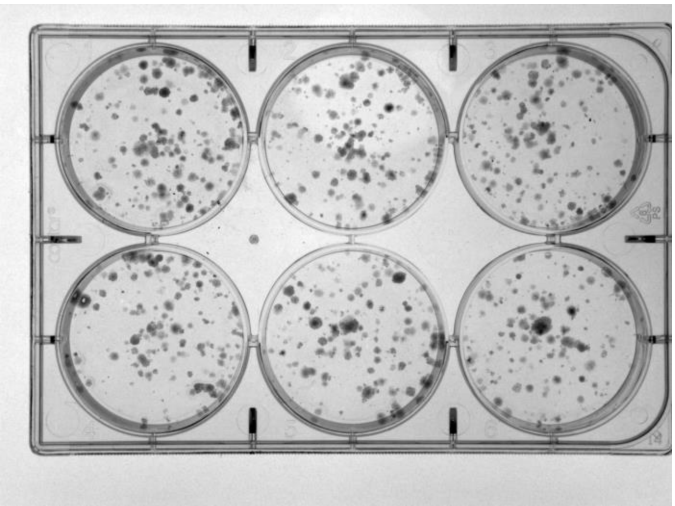

-Doxy

+Doxy

SY5Y sh#1 RUN 1

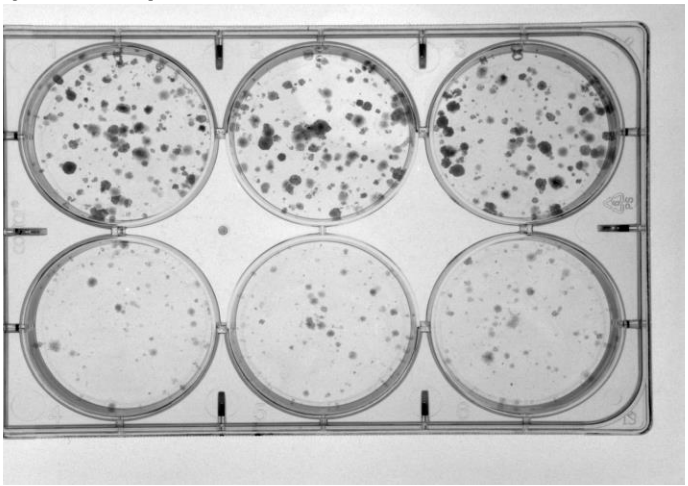

-Doxy

+Doxy

SY5Y sh#2 RUN 1

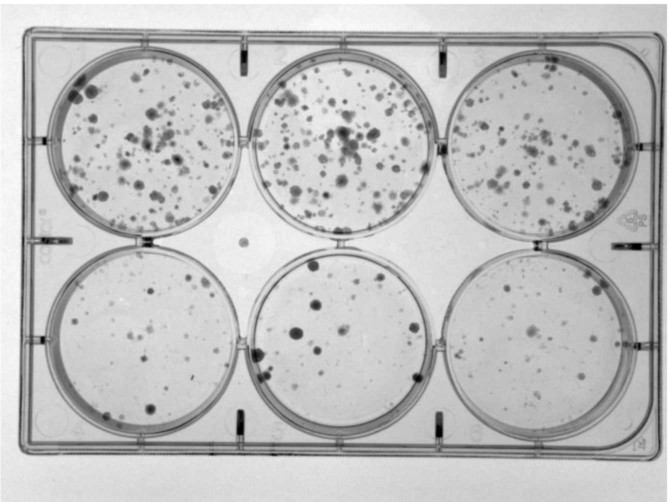

-Doxy

+Doxy

Supp Figure 4b
SY5Y shRNA colonies

SY5Y FH1UTG RUN 2

-Doxy

+Doxy

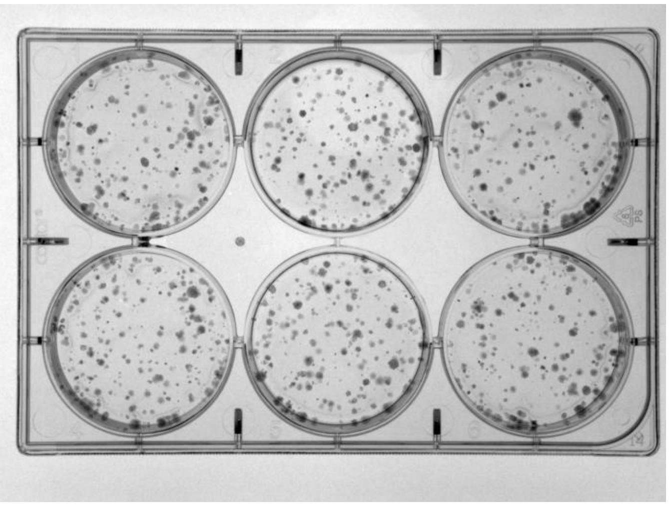

SY5Y sh#1 RUN 2

-Doxy

+Doxy

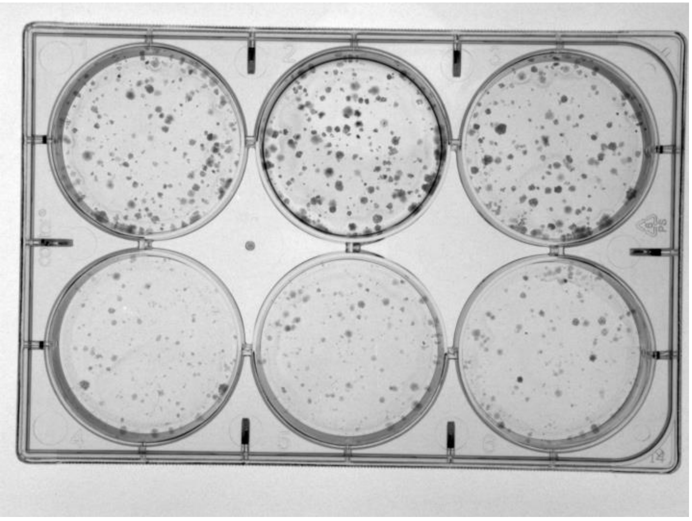

SY5Y sh#2 RUN 2

-Doxy

+Doxy

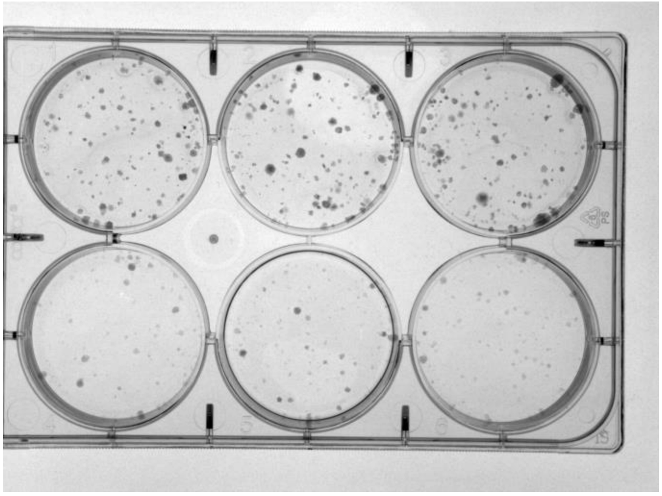

Supp Figure 4b
SY5Y shRNA colonies

SY5Y FH1UTG RUN 3

-Doxy

+Doxy

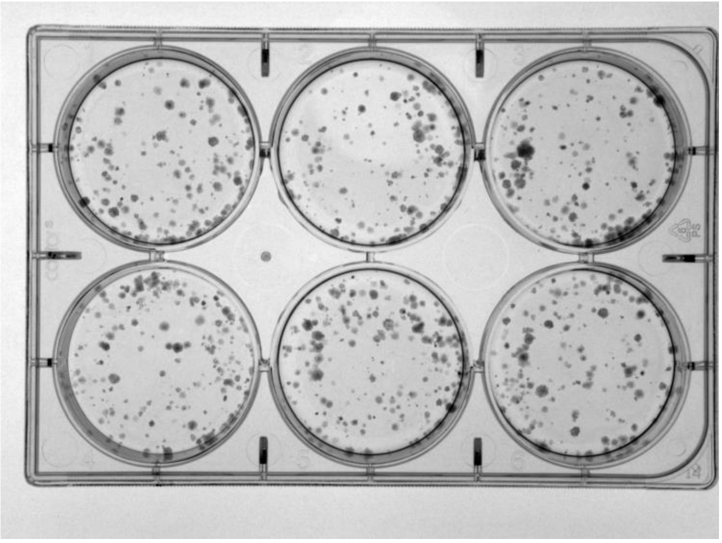

SY5Y sh#1 RUN 3

-Doxy

+Doxy

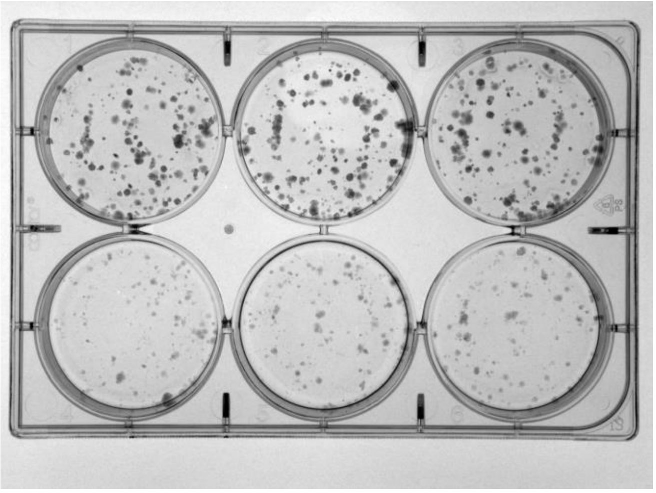

SY5Y sh#2 RUN 3sad

-Doxy

+Doxy

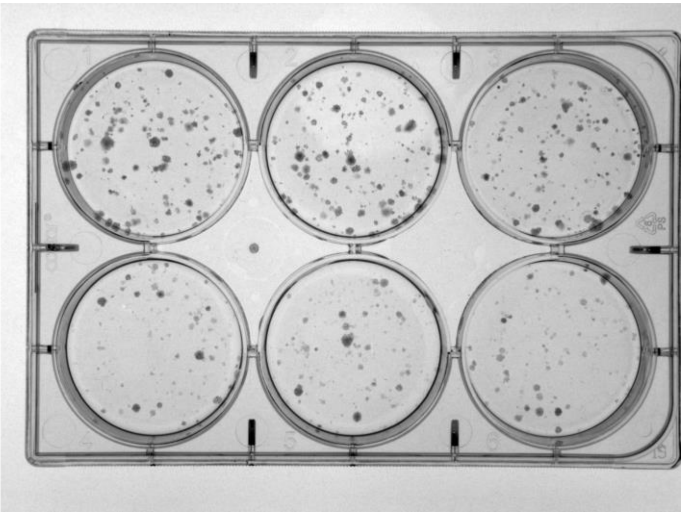

Supp Figure 5c

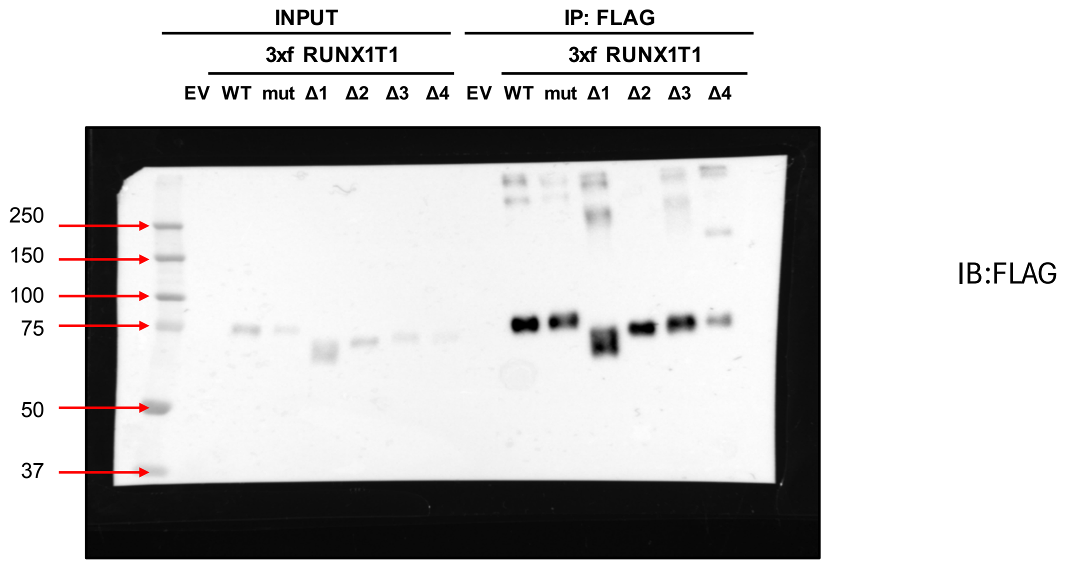

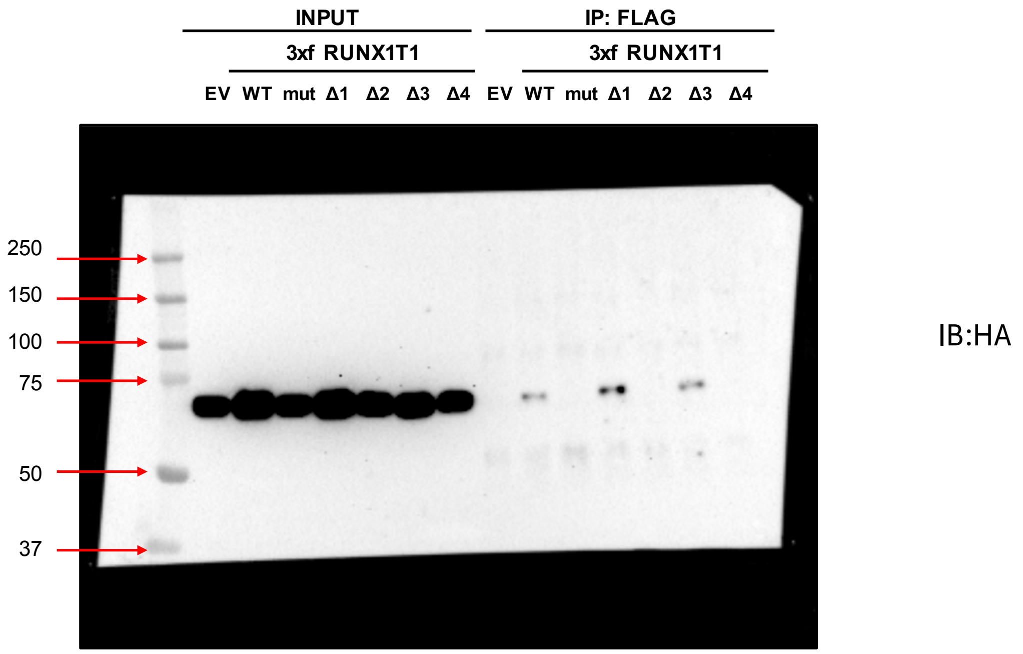

Supp Figure 5d

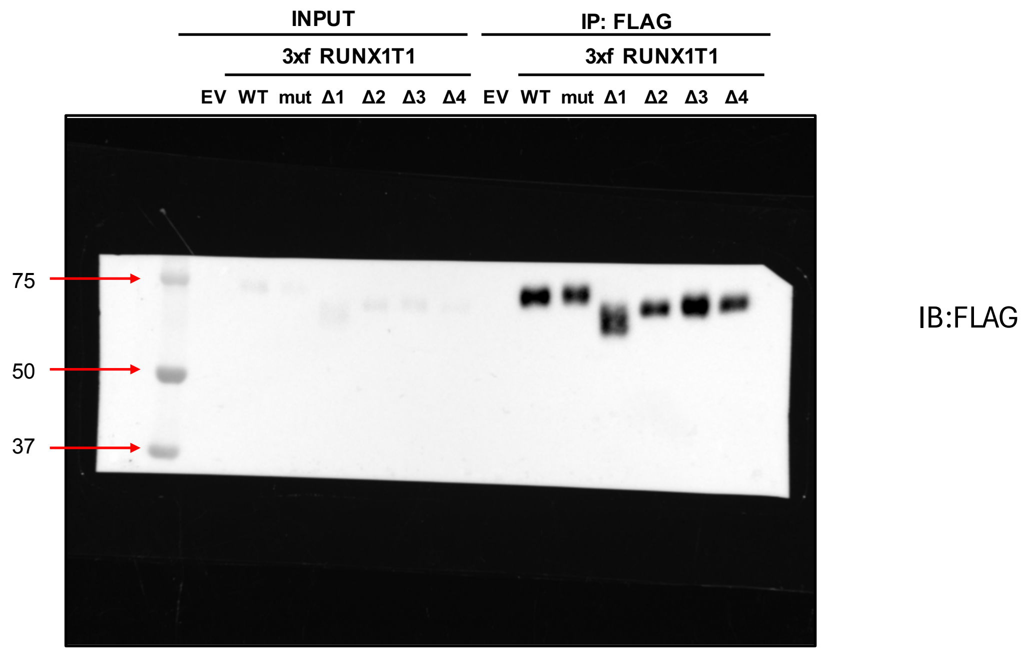

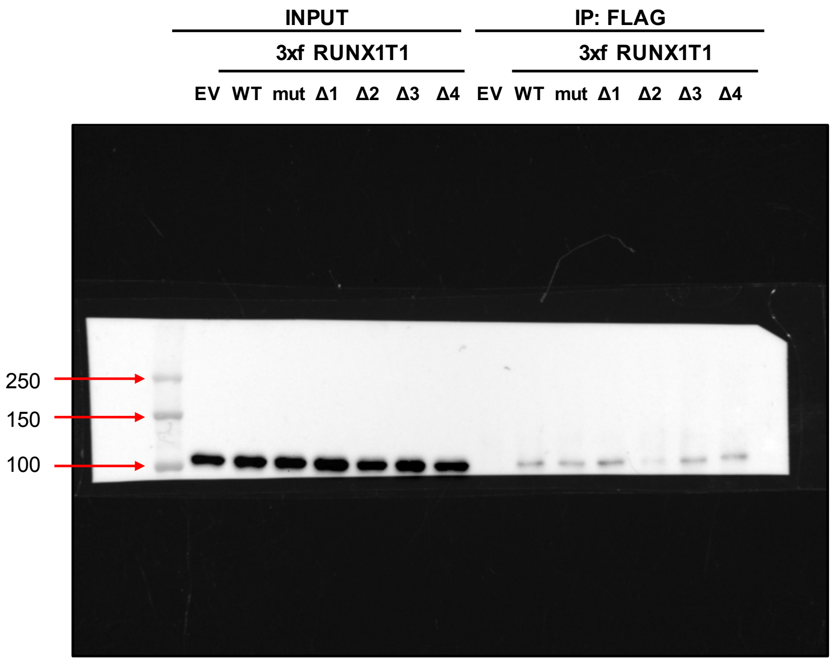

ì

Supp Figure 5e
KELLY shHAND2

Run 1_main

Ladder
RUNX1T1_NODOX
RUNX1T1_DOX

250kD
150kD
100kD
75kD
50kD

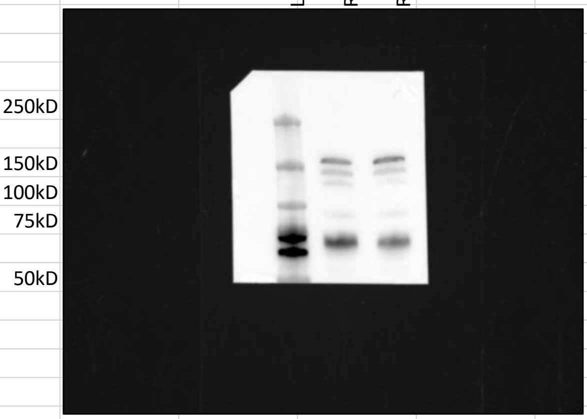

Ladder
HAND2_NODOX
HAND2_DOX

37kD
25kD
20kD
15kD
10kD

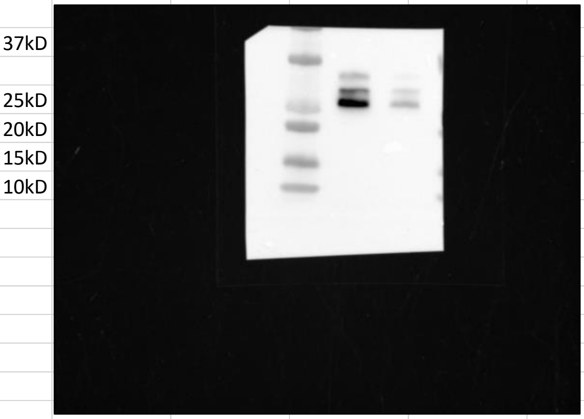

Ladder
GAPDH_NODOX
GAPDH_DOX

37kD
25kD
20kD
15kD
10kD

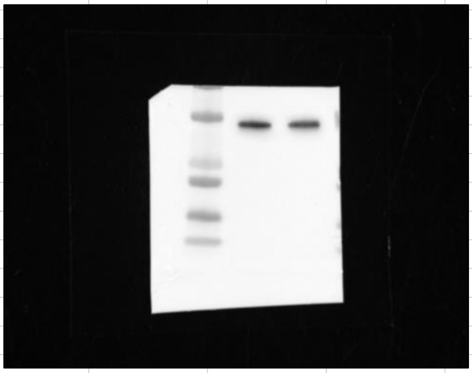

Supp Figure 5e continued
KELLY shHAND2

Run 2

Ladder
RUNX1T1_NODOX
RUNX1T1_DOX

250kD
150kD
100kD
75kD
50kD

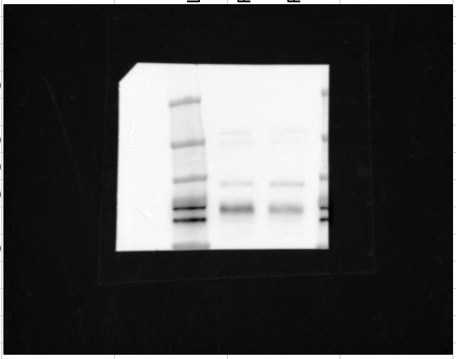

Ladder
HAND2_NODOX
HAND2_DOX

37kD
25kD
20kD
15kD
10kD

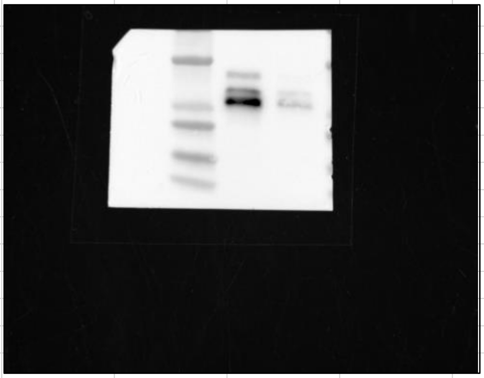

Ladder
GAPDH_NODOX
GAPDH_DOX

37kD
25kD
20kD
15kD
10kD

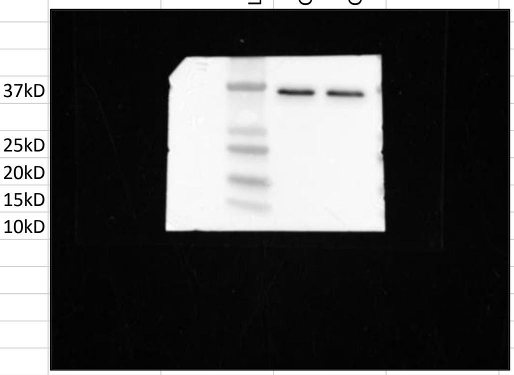

Supp Figure 5e continued
KELLY shHAND2

Run 3

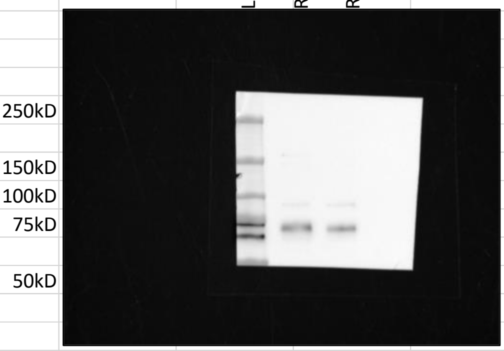

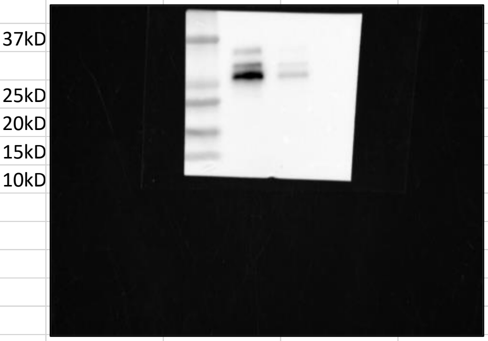

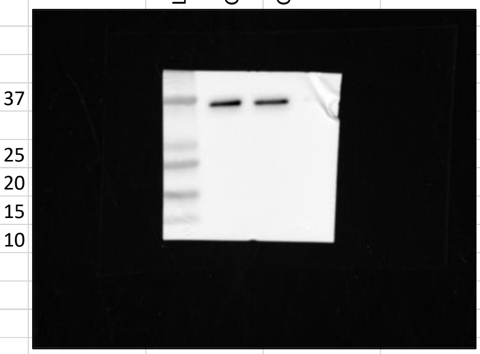

Supp Figure 5g

	Input	Input_HAND2-KD	HAND2 ChIP	HAND2 CHIP-HAND2-KD	RUNX1T1 ChIP	RUNXT1 ChIP-HAND2-KD
ETO_peak1-rep1	1	1	47.17	13.25	14.35	4.2
ETO_peak1-rep2	1	1	53.77	11.98	14.89	6.92
ETO_peak1-rep3	1	1	33.84	15.38	11.49	6.4
ETO_peak2-rep1	1	1	18.65	3.76	5.71	1.72
ETO_peak2-rep2	1	1	17.64	1.75	5.38	2.5
ETO_peak2-rep3	1	1	14.73	4.65	5.25	2.09
ETO_peak3-rep1	1	1	54.52	10.66	18.52	4.39
ETO_peak3-rep2	1	1	43.07	12.4	20.82	6.19
ETO_peak3-rep3	1	1	48.39	17.9	25.43	8.84
ETO_peak4-rep1	1	1	13.55	2.78	3.55	1.02
ETO_peak4-rep2	1	1	10.57	3.24	3.72	1.52
ETO_peak4-rep3	1	1	11.4	4.93	4.12	2.91
	Normalized Fold Enrichment 2^-DDCT					

OFFICIAL NAME	Lab. TUBE NAME	Sequence_FW	Sequence_RV	Coordinates on hg38
**ETO_Peak1**	1971	ACTTGCACAAACCAGAGCTG	GGACTTCCGCCTTGTAATGA	chr8:143039154+143039227
**ETO_Peak2**	2692	AGGGCGTCATCCTCTTCTTG	TGGCCAGGCTTTTATTGTGC	chr17:35801573+35801650
**ETO_Peak3**	2746	AATCCCAACCCCCGCATATG	AAGGCTGCATGGAGAAAAGC	chr15:62878257+62878379
**ETO_Peak4**	ngfr	ACACACACACATACACACCA	CTGCCCACGAGAGCTACAAG	chr17:49515378+49515507

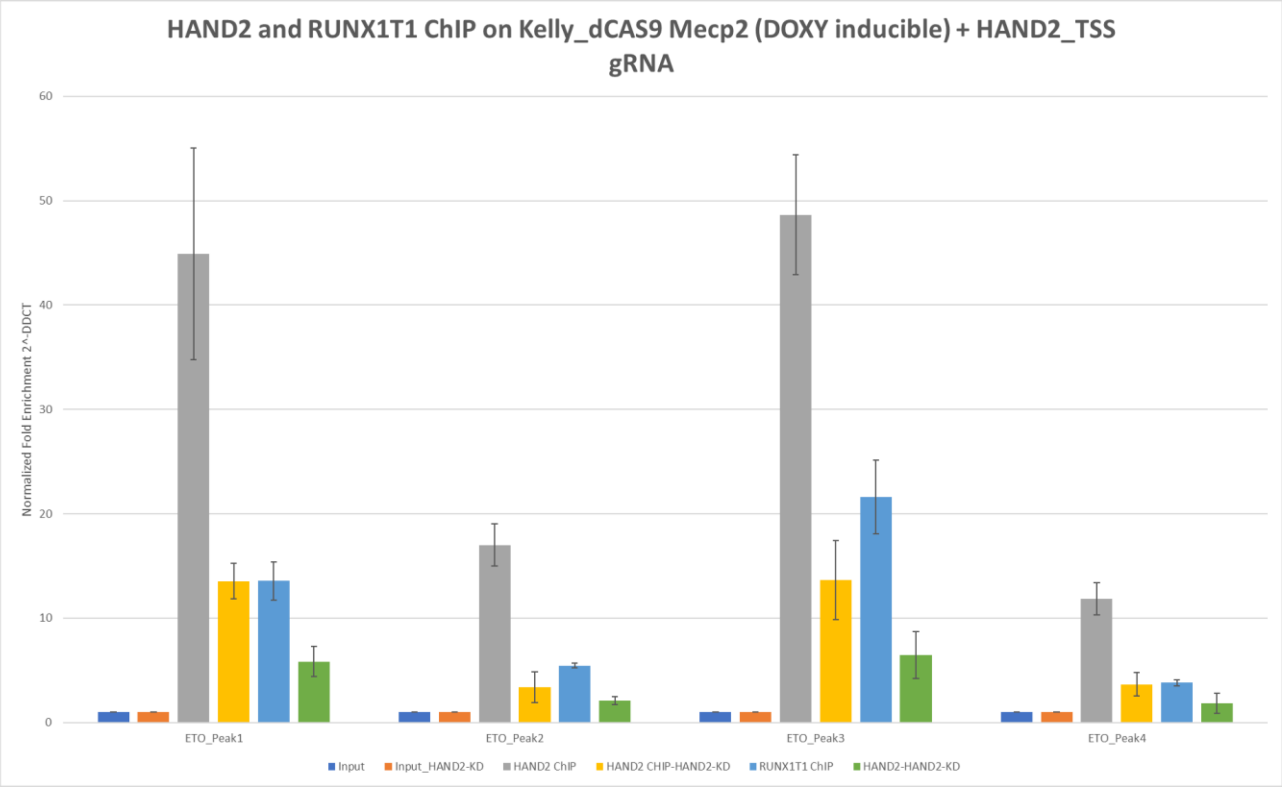

	Two-tailed P value		
	HAND2	RUNX1T1	
**ETO_Peak1**	0.0062 **	0.0045 **	*≦ 0,05
**ETO_Peak2**	0.0007 ***	0.0002 ***	**≦ 0,01
**ETO_Peak3**	0.0009 ***	0.0033 **	***≦ 0,001
**ETO_Peak4**	0.0018 **	0.0284 *	

Supp Figure 7b
RH41 MYCN Knockdown RUNX1T1 and GAPDH

RH41 cells
15/02/2023

Merged with protein marker

Anti-Runx1t1

gel#1

gel#2

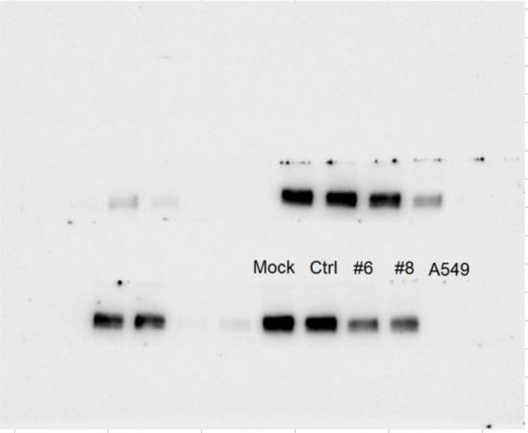

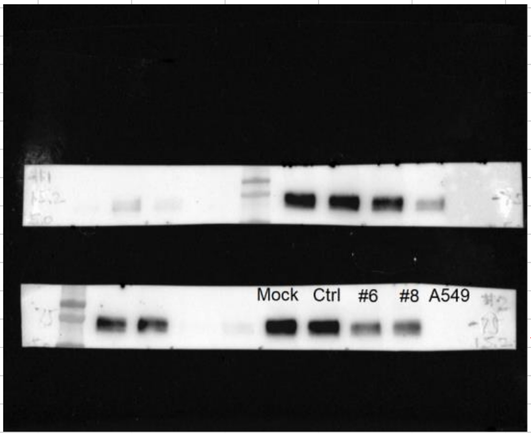

75kD
50kD

Anti-GAPDH

gel#1

gel#2

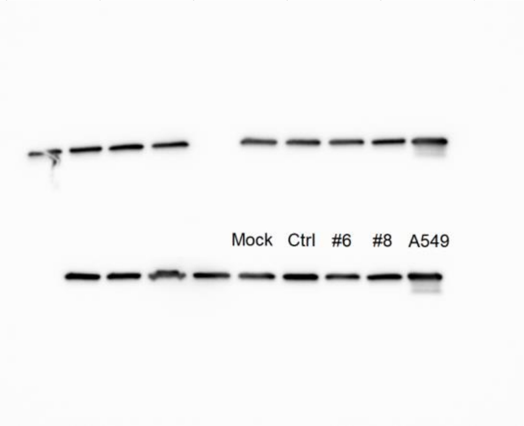

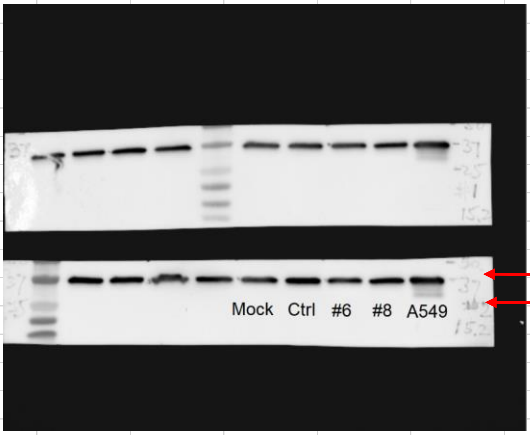

37kD
25kD

Note: Ctrl, siRNA#6 and #8 in gel#2 were used for supplementary Fig.7B

Supp Figure 7b
RH41 MYCN Knockdown RUNX1T1 IncuCyte

Run#1 **Set up on**
10/11/2022

Elapsed (hrs)	control siRNA	control siRNA	Ctrl siRNA Average	% of Ctrl at 0 h	siRNA #8	siRNA #8	siRNA#8Average	% of Ctrl siRNA at 0 h
0.00	707.67	669.89	688.78	**100.0%**	506.22	518.56	512.39	74.4%
12.00	952.67	865.78	909.22	132.0%	605.89	606.00	605.94	88.0%
24.00	1163.67	1113.56	1138.61	165.3%	731.00	746.22	738.61	107.2%
36.00	1442.44	1377.56	1410.00	204.7%	885.22	884.67	884.94	128.5%
48.00	1791.78	1722.44	1757.11	255.1%	1042.89	1036.89	1039.89	151.0%
60.00	2313.56	2133.33	2223.44	322.8%	1226.56	1226.22	1226.39	178.1%
72.00	2645.33	2578.67	2612.00	379.2%	1434.11	1444.78	1439.44	209.0%
84.00	2896.00	2768.67	2832.33	411.2%	1732.11	1756.22	1744.17	253.2%
96.00	2740.89	2661.11	2701.00	392.1%	2168.56	2131.22	2149.89	312.1%

Run#2 **Set up on**
12/12/2022

					% of Ctrl siRNA at 0 h			
**Elapsed (hrs)**	**Mock**	**Ctrl siRNA**	**siRNA #6**	**siRNA #8**	**Mock**	**Ctrl siRNA**	**siRNA #6**	**siRNA #8**
0.00	504.2	374.9	332.4	261.6	134.50	**100.00**	88.68	69.77
12.03	632.3	458.9	391.3	296.8	168.67	122.41	104.39	79.16
24.03	849.4	585.0	423.3	357.6	226.59	156.05	112.92	95.38
36.03	1333.7	947.1	532.9	603.6	355.75	252.64	142.15	161.00
48.03	1613.9	1171.0	573.6	711.1	430.50	312.36	152.99	189.69
60.03	1852.0	1415.0	634.6	750.1	494.01	377.45	169.26	200.09
72.03	2202.0	1508.1	613.8	723.9	587.37	402.28	163.72	193.09
84.03	2313.6	1823.8	673.4	868.1	617.13	486.48	179.64	231.56
96.03	1947.7	2039.1	789.0	987.1	519.53	543.92	210.46	263.31

Run#3 **Set up on**
6/02/2023 **2/02/2023**

					% of Ctrl siRNA at 0 h			
**Elapsed (hrs)**	**Mock**	**Ctrl siRNA**	**siRNA #6**	**siRNA #8**	**Mock**	**Ctrl siRNA**	**siRNA #6**	**siRNA #8**
0	803.6	645.3	429.0	518.1	125%	**100%**	66%	80%
12	1044.2	773.4	467.1	545.4	162%	120%	72%	85%
24	1429.1	993.3	558.3	711.0	221%	154%	87%	110%
36	1843.7	1284.0	565.6	818.6	286%	199%	88%	127%
48	2278.1	1624.6	604.4	981.3	353%	252%	94%	152%
60	2750.3	2025.8	668.1	1158.3	426%	314%	104%	179%
72	2668.3	2465.6	758.3	1366.8	413%	382%	118%	212%
84	2460.6	2898.3	823.3	1649.7	381%	449%	128%	256%
96	2266.1	2919.0	1078.8	2001.0	351%	452%	167%	310%

Supp Figure 7c
SCLC cell lines RUNX1T1 and ACTIN

Western blot
25/08/2020

gel#2
Anti-Runx1t1

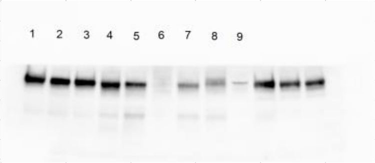

Lane	
	DMS-53
	DMS-273
	DMS-454
	H69
	H889
	A549
	SH-SY5Y
	Kelly
9	protein marker

Lane 1-7 for Supplementary Figure 7C

Western blot
25/08/2020

gel#2
Anti-Runx1t1

Blots merged with protein marker

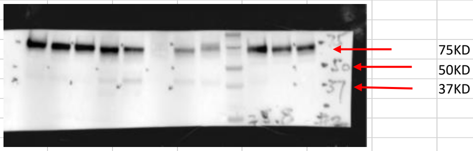

75KD
50KD
37KD

Anti-Actin
gel#2

gel#3

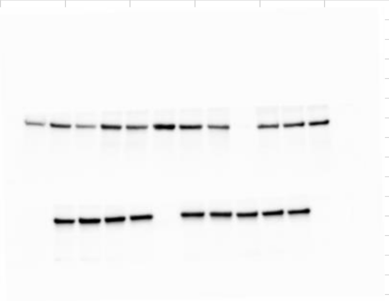

Note: gel#2 is for Supplementary Fig.7C

Anti-Actin
gel#2

gel#3

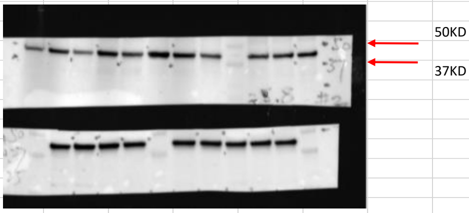

50KD
37KD

Source Data
Supp Figure 7d DMS-273 with RUNX1T1 and ACTIN

DMS-273 cells
16/03/2023
gel#1
Anti-Runx1t1
Doxy

75KD

sh EV sh #1 sh #2
\- + - + - +

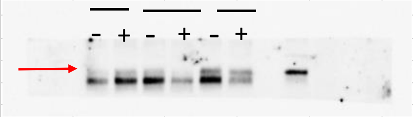

Anti-GAPDH
37KD
25KD

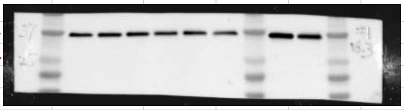

Blot merged with protein marker

DMS-273 cells
15/03/2023
Doxy

sh EV sh #1 sh #2
\- + - + - + Kelly

Anti-Runx1t1 gel#2
75KD

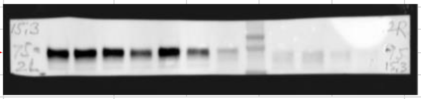

Blot merged with protein marker
Labeled samples in gel#2 used for Supplementary figure7D

Anti-GAPDH

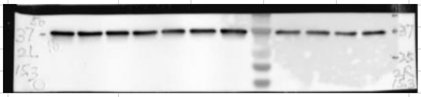

Blot merged with protein marker

Supp Figure 7d
DMS-273 shRNA RUNX1T1 colonies

Run 1

1000 cells/well

DMS-273-FHIUTG

Doxy (-)

Doxy (+)

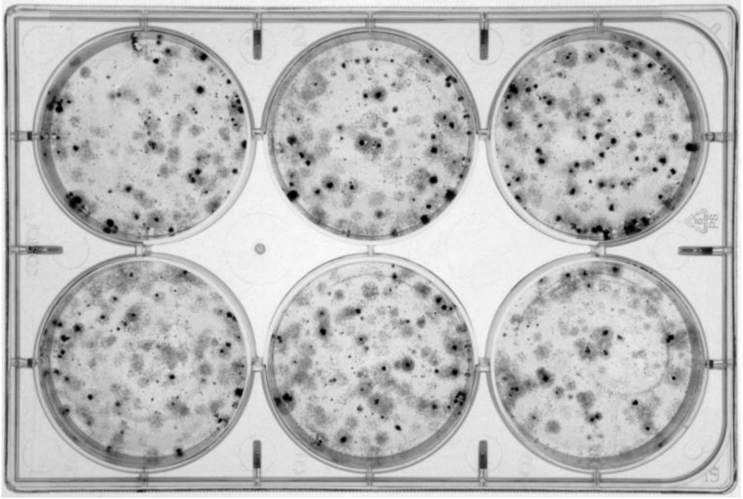

DMS-273-Sh#1

Doxy (-)

Doxy (+)

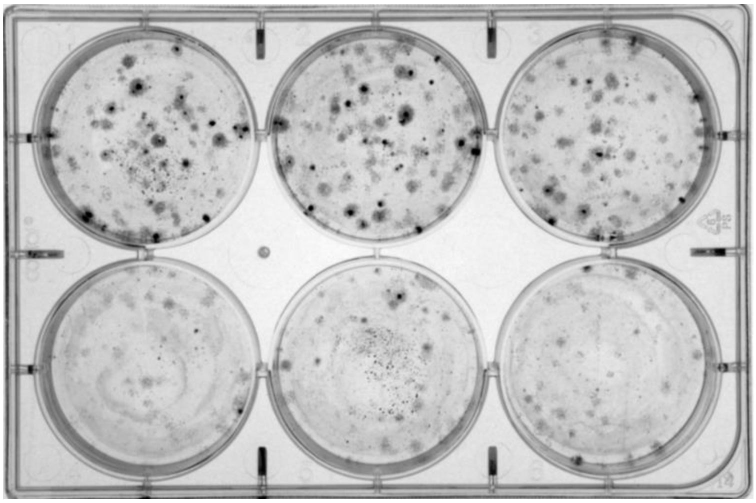

DMS-273-Sh#2

Doxy (-)

Doxy (+)

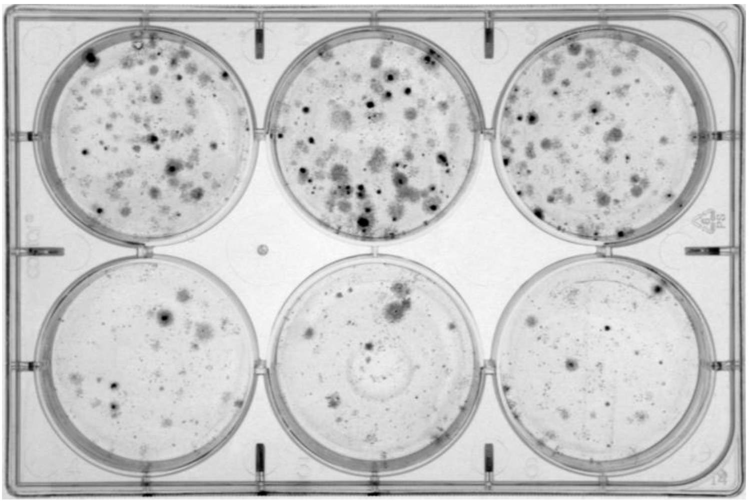

Supp Figure 7d
DMS-273 shRNA RUNX1T1 colonies

Run 2

1000 cells/well

DMS-273-FHIUTG

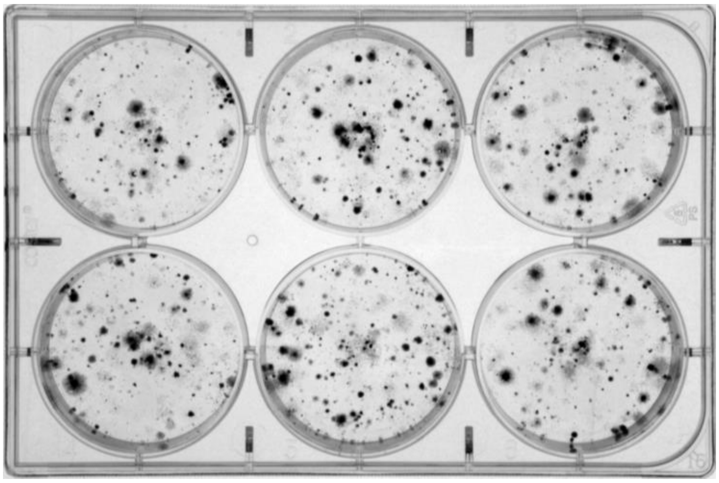

Doxy (-)

Doxy (+)

DMS-273-Sh#1

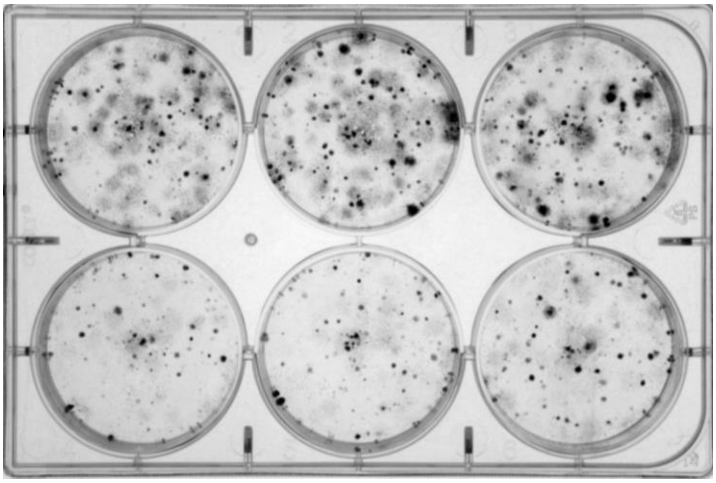

Doxy (-)

Doxy (+)

DMS-273-Sh#2

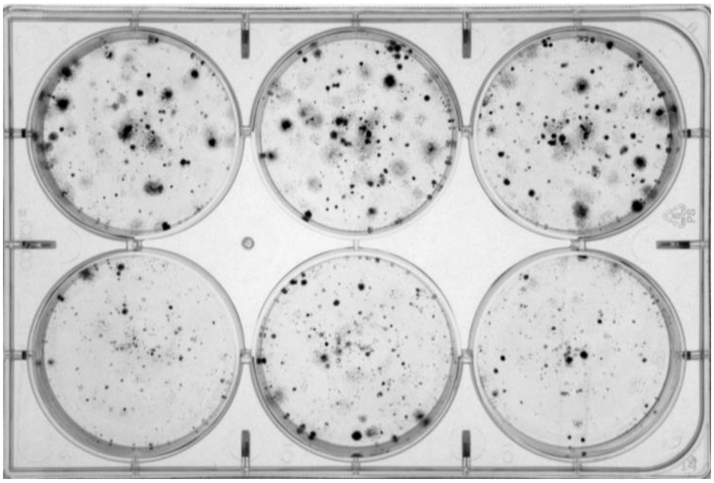

Doxy (-)

Doxy (+)

Supp Figure 7d
DMS-273 shRNA RUNX1T1 colonies

Run 3

1000 cells/well

DMS-273-FHIUTG

Doxy (-)

Doxy (+)

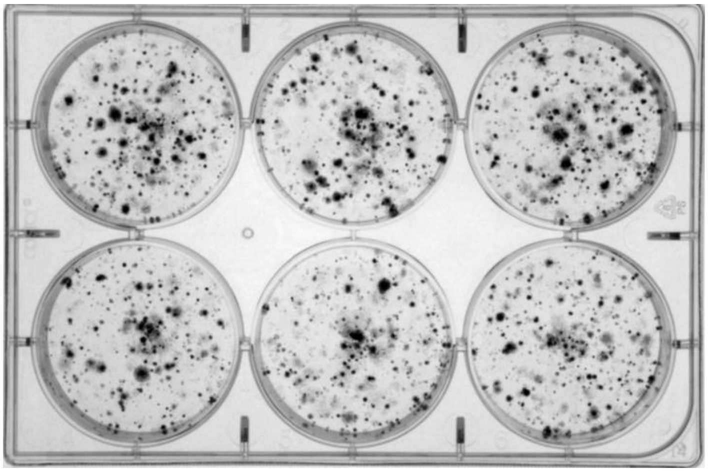

DMS-273-Sh#1

Doxy (-)

Doxy (+)

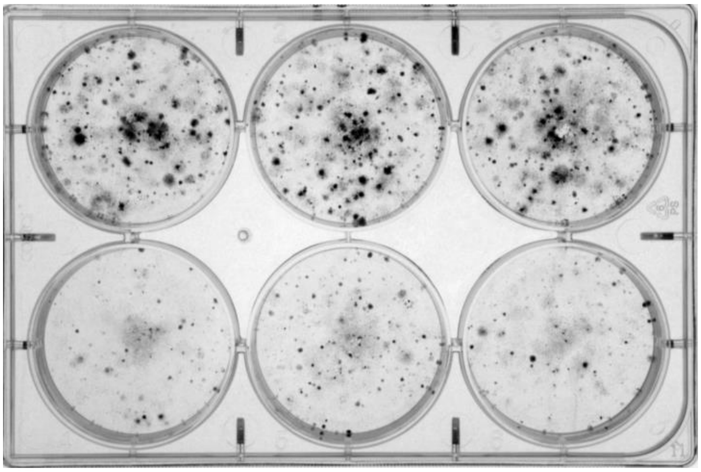

DMS-273-Sh#2

Doxy (-)

Doxy (+)

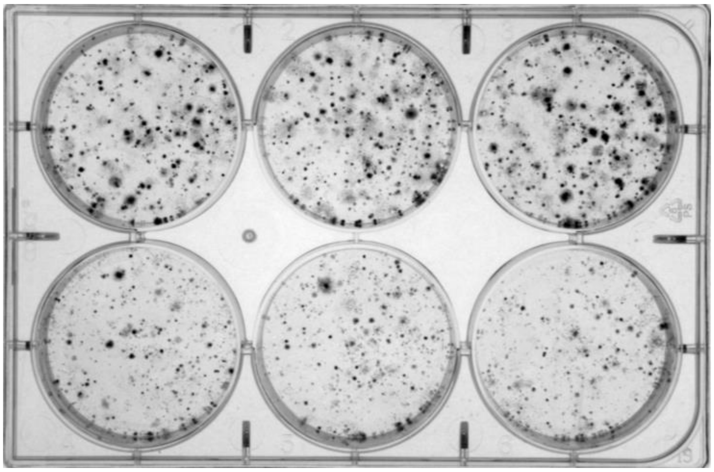

Supp Figure 7e
DMS-53 with RUNX1T1 and ACTIN

DMS-53 cells

17/12/2021

sh EV sh #1 sh #2

Doxy - + - + - +

Anti-Runx1t1

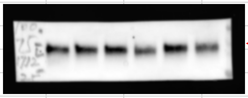

75KD

50KD

Anti-GAPDH

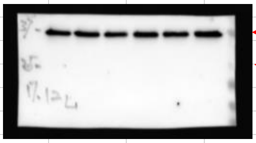

37KD

25KD

Blot merged with protein marker

DMS-53 cells

7/03/2023

gle#2

sh EV sh #1 sh #2

Doxy - + - + - +

Anti-Runx1t1

Anti-GAPDH

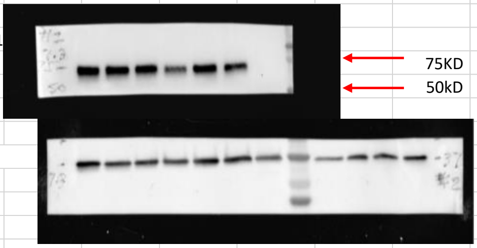

75KD

50kD

37KD

Blot merged with protein marker

DMS-53 cells

7/03/2023

gle#1

sh EV sh #1 sh #2

Doxy - + - + + -

Anti-Runx1t1

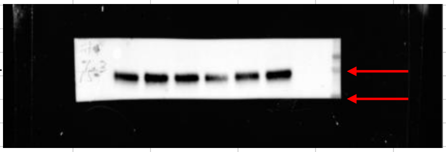

75KD

50kD

Note: For sh#2, Doxy(+) sample was accidentally loaded on the left side of the Doxy(-) sample.

sh EV sh #1 sh #2

Doxy - + - + + -

Anti-GAPDH

37KD

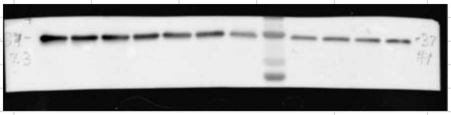

Blot merged with protein marker

Supp Figure 7e
DMS-53 with shRNA RUNX1T1 colonies

Run1

500 cells/well DMS-53/FH1UTG

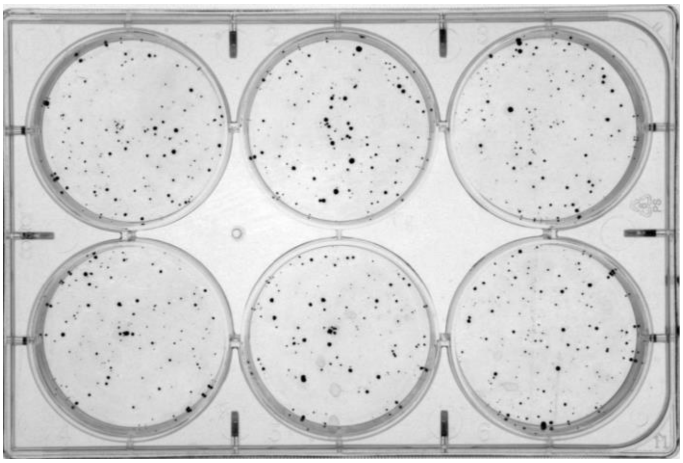

Doxy (-)

Doxy (+)

DMS-53/sh#1

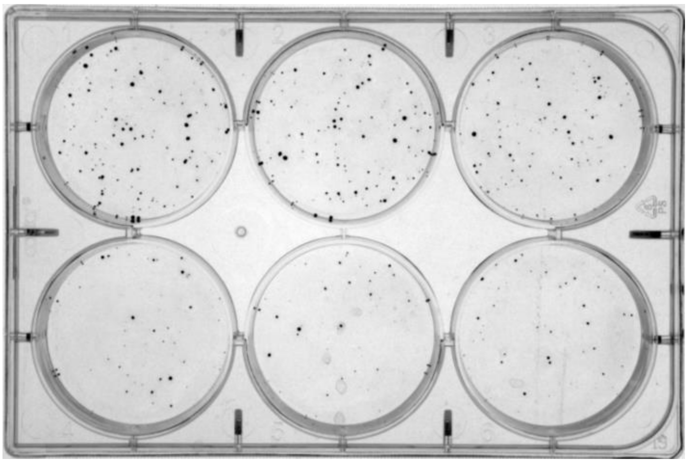

Doxy (-)

Doxy (+)

DMS-53/sh#2

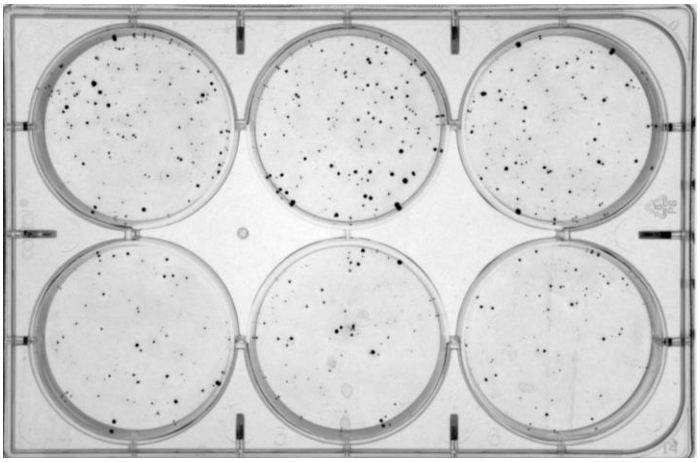

Doxy (-)

Doxy (+)

Supp Figure 7e
DMS-53 with shRNA RUNX1T1 colonies

Run 2

500 cells/well DMS-53/FH1UTG

Doxy (-)

Doxy (+)

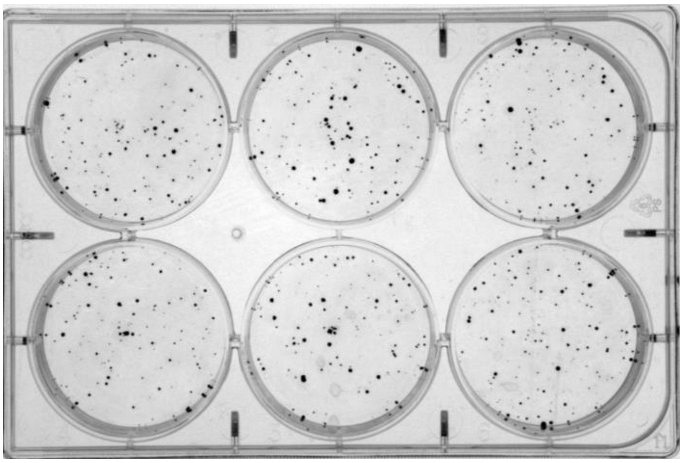

DMS-53/sh#1

Doxy (-)

Doxy (+)

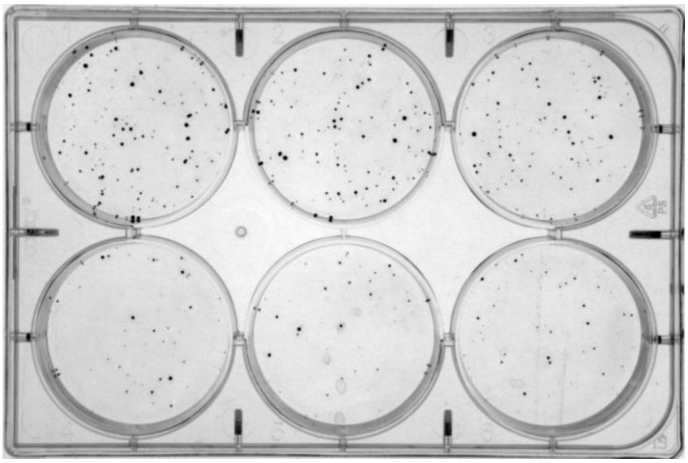

DMS-53/sh#2

Doxy (-)

Doxy (+)

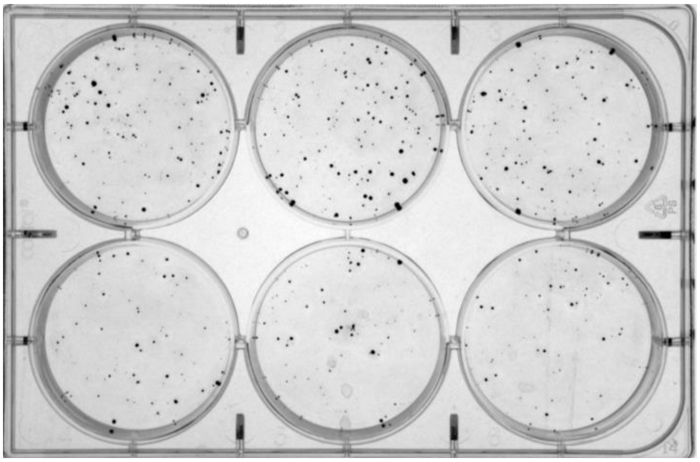

Supp Figure 7e
DMS-53 with shRNA RUNX1T1 colonies

Run 3

500 cells/well DMS-53/FH1UTG

Doxy (-)

Doxy (+)

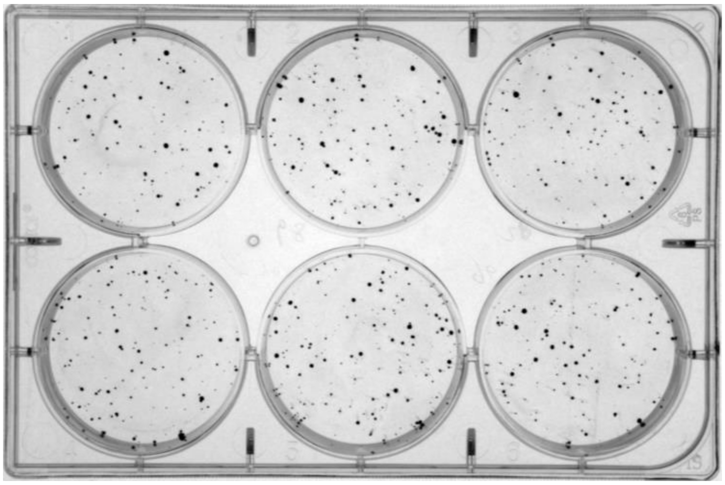

DMS-53/sh#1

Doxy (-)

Doxy (+)

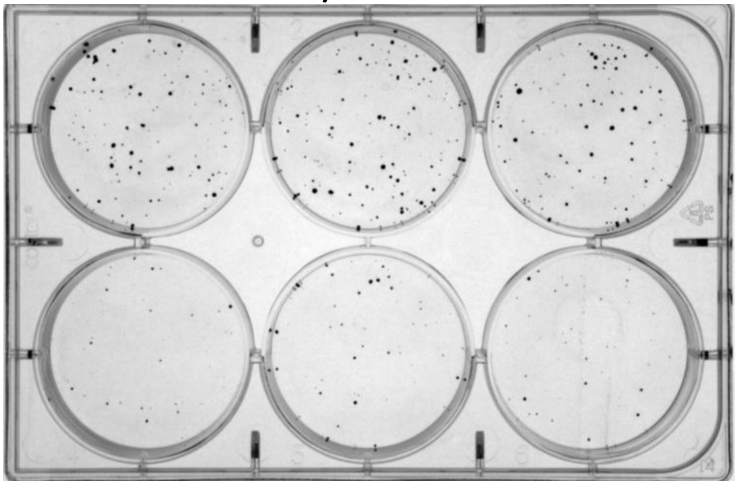

DMS-53/sh#2

Doxy (-)

Doxy (+)

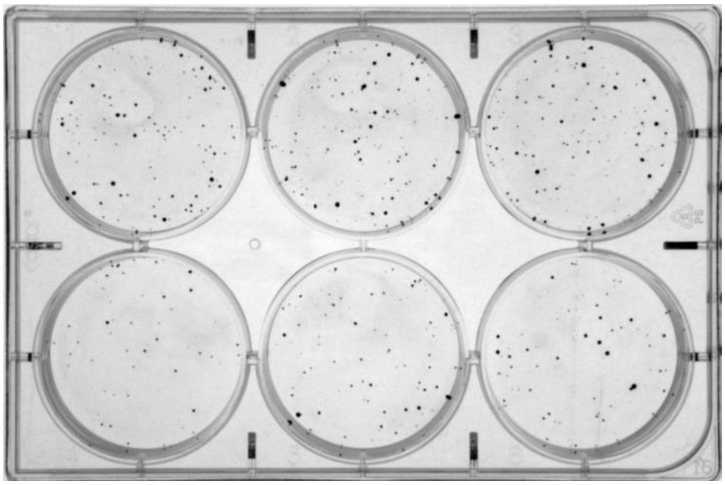

Supplement: Supplementary file 1 — Supplementary Information [file 41467_2024_49871_MOESM1_ESM.pdf]
