# Supplementary material for: The transcriptional co-repressor Runx1t1 is essential for MYCN-driven neuroblastoma tumorigenesis
Source: Nat Commun. 2024 Jul 11;15:5585. doi: 10.1038/s41467-024-49871-0 (PMC11239676; doi:10.1038/s41467-024-49871-0)
Supplement: Supplementary file 3 — Reporting Summary [file 41467_2024_49871_MOESM3_ESM.pdf]

# nature portfolio

Corresponding author(s): Murray Norris

Last updated by author(s): Mar 9, 2024

# Reporting Summary

Nature Portfolio wishes to improve the reproducibility of the work that we publish. This form provides structure for consistency and transparency in reporting. For further information on Nature Portfolio policies, see our Editorial Policies and the Editorial Policy Checklist.

## Statistics

For all statistical analyses, confirm that the following items are present in the figure legend, table legend, main text, or Methods section.

| n/a | Confirmed | |
|---|---|---|
| ☐ | ☒ | The exact sample size (*n*) for each experimental group/condition, given as a discrete number and unit of measurement |
| ☐ | ☒ | A statement on whether measurements were taken from distinct samples or whether the same sample was measured repeatedly |
| ☐ | ☒ | The statistical test(s) used AND whether they are one- or two-sided<br>*Only common tests should be described solely by name; describe more complex techniques in the Methods section.* |
| ☐ | ☒ | A description of all covariates tested |
| ☐ | ☒ | A description of any assumptions or corrections, such as tests of normality and adjustment for multiple comparisons |
| ☐ | ☒ | A full description of the statistical parameters including central tendency (e.g. means) or other basic estimates (e.g. regression coefficient) AND variation (e.g. standard deviation) or associated estimates of uncertainty (e.g. confidence intervals) |
| ☒ | ☐ | For null hypothesis testing, the test statistic (e.g. *F*, *t*, *r*) with confidence intervals, effect sizes, degrees of freedom and *P* value noted<br>*Give P values as exact values whenever suitable.* |
| ☒ | ☐ | For Bayesian analysis, information on the choice of priors and Markov chain Monte Carlo settings |
| ☐ | ☒ | For hierarchical and complex designs, identification of the appropriate level for tests and full reporting of outcomes |
| ☐ | ☒ | Estimates of effect sizes (e.g. Cohen's *d*, Pearson's *r*), indicating how they were calculated |

*Our web collection on statistics for biologists contains articles on many of the points above.*

## Software and code

Policy information about availability of computer code

| | |
|---|---|
| Data collection | No software was used |
| Data analysis | RNA-seq: fastQC (v0.11.5), STAR (v2.5), samtools (v1.3.1), RSEM (v1.2.31)<br>ChIP-seq: Bowtie2 (v2.1.0), samtools (v1.9), MACS2 (v2.1.1), BEDTools (v2.26.0), R (v4.1.2), deeptools (v3.5.1). ChIP-seq analysis code can be found in the following public GitHub repository: https://github.com/CCI-BIO/RUNX1T1 |

For manuscripts utilizing custom algorithms or software that are central to the research but not yet described in published literature, software must be made available to editors and reviewers. We strongly encourage code deposition in a community repository (e.g. GitHub). See the Nature Portfolio guidelines for submitting code & software for further information.

## Data

Policy information about availability of data

All manuscripts must include a data availability statement. This statement should provide the following information, where applicable:

- Accession codes, unique identifiers, or web links for publicly available datasets
- A description of any restrictions on data availability
- For clinical datasets or third party data, please ensure that the statement adheres to our policy

All data are available within the Article and Supplementary Files, or available from the corresponding authors on reasonable request. The RNA-sequencing and ChIP-sequencing data generated in this study have been deposited in the GEO database under accession code GSE230265 https://www.ncbi.nlm.nih.gov/geo/query/

acc.cgi?acc=GSE230265. The processed ChIP-sequencing and RNA-sequencing data are available at GEO database under accession code GSE230265 and in our github repository at https://github.com/CCI-bio/RUNX1T1. The ChIPseq dataset of MYCN-amplified Neuroblastoma cell lines data used in this study are available in the GEO database under accession code GSE94824 https://www.ncbi.nlm.nih.gov/geo/query/acc.cgi?acc=GSE94824. The mass spectrometry proteomics data have been deposited to the ProteomeXchange Consortium via the PRIDE partner repository with the dataset identifier PXD050375. The ChIP-sequencing and RNA-sequencing data can also be explored/analysed directly via the R2 genomics analysis and visualization platform (https://r2.amc.nl).

## Research involving human participants, their data, or biological material

Policy information about studies with human participants or human data. See also policy information about sex, gender (identity/presentation), and sexual orientation and race, ethnicity and racism.

| | |
|---|---|
| Reporting on sex and gender | Publicly available databases were used for RNA expression cohorts and Cell Line databases. For tissue microarrays, sex and gender information were not collected. |
| Reporting on race, ethnicity, or other socially relevant groupings | N/A |
| Population characteristics | N/A |
| Recruitment | For tissue microarrays, formalin fixed paraffin embedded samples consented for use in research were used |
| Ethics oversight | Sydney Children's Hospital Network, Sydney Australia |

Note that full information on the approval of the study protocol must also be provided in the manuscript.

# Field-specific reporting

Please select the one below that is the best fit for your research. If you are not sure, read the appropriate sections before making your selection.

- [x] Life sciences
- [ ] Behavioural & social sciences
- [ ] Ecological, evolutionary & environmental sciences

For a reference copy of the document with all sections, see nature.com/documents/nr-reporting-summary-flat.pdf

# Life sciences study design

All studies must disclose on these points even when the disclosure is negative.

| | |
|---|---|
| Sample size | At least three independent biological replicates were used in each experiment. |
| Data exclusions | No data were excluded from the analysis |
| Replication | All attempts at replication were successful |
| Randomization | Mice were randomly assigned to doxycycline or control food once their tumours reached 100mm3 |
| Blinding | Blinding in the doxycycline experiments was not possible as the doxycycline food is coloured red |

# Reporting for specific materials, systems and methods

We require information from authors about some types of materials, experimental systems and methods used in many studies. Here, indicate whether each material, system or method listed is relevant to your study. If you are not sure if a list item applies to your research, read the appropriate section before selecting a response.

## Materials & experimental systems

| n/a | Involved in the study | |
|---|---|---|
| ☐ | ☒ | Antibodies |
| ☐ | ☒ | Eukaryotic cell lines |
| ☒ | ☐ | Palaeontology and archaeology |
| ☐ | ☒ | Animals and other organisms |
| ☐ | ☒ | Clinical data |
| ☒ | ☐ | Dual use research of concern |
| ☒ | ☐ | Plants |

## Methods

| n/a | Involved in the study | |
|---|---|---|
| ☐ | ☒ | ChIP-seq |
| ☒ | ☐ | Flow cytometry |
| ☒ | ☐ | MRI-based neuroimaging |

## Antibodies

**Antibodies used**

RUNX1T1 (rabbit polyclonal, 15494-1-AP, Proteintech)
MYCN (mouse monoclonal B8.4.B, sc-53993, Santa Cruz)
GAPDH (mouse monoclonal G-9, sc-365062, Santa Cruz)
ACTIN (rabbit polyclonal antibody, A2066, Sigma-Aldrich)
MAX (rabbit monoclonal S-20, 4739S, Cell Signalling)
MXD1 (rabbit polyclonal, 19547-1-AP, Proteintech)
MXD2 (rabbit polyclonal, A12098, Abclonal)
MXD3 (mouse polyclonal, 249041, United States Biological)
MXD4 (rabbit polyclonal, ab220495, Abcam)
MGA (MXD5) (mouse monoclonal MGA6A4H5, sc-81105, Santa Cruz)
MNT (MXD6) (rabbit polyclonal, MBS9606130, MyBioSource)
MLX (MXD7) (mouse monoclonal F-12, sc-393086, Santa Cruz)
MYCN (mouse monoclonal NCM II 100, ab16898, Abcam)
MYCN (rabbit polyclonal, 10159-2-AP, Proteintech)
BIII tubulin (802001, Biolegend)
GAPDH (mouse monoclonal G-9, sc-365062, Santa Cruz)
FLAG (mouse monoclonal M2, F3165, Sigma Aldrich)
IgG (mouse monoclonal, sc-2025, Santa Cruz)
Anti-HA (rabbit polyclonal, ab9110, ABcam)
HAND2 (Rabbit Monoclonal EPR19451, Abcam)

**Validation**

We have been studying MYCN in childhood neuroblastoma for over two decades with numerous publications detecting this protein using primary tumor material as well as cell lines involving knock down and overexpression. MYCN was also validated using the doxycyline-inducible SH-EP 21N cells. Likewise, RUNX1T1 and MYCN binding partners were validated using knock down studies.

## Eukaryotic cell lines

Policy information about cell lines and Sex and Gender in Research

**Cell line source(s)**

Kelly, DMS-273 and DMS-53 were obtained from European Collection of Authenticated Cell Lines via Cell Bank Australia. BE (2)-C and SH-SY5Y were obtained from June Biedler at Memorial Sloan Kettering Cancer Center. Rh3 and Rh41 were obtained from Peter Houghton, Greehey Children's Cancer Research Institute, USA. SH-EP 21N cells were obtained from Manfred Schwab, German Cancer Research Center, Heidelberg, Germany.

**Authentication**

STR profiling was used to authenticate the cell lines by Cell Bank Australia

**Mycoplasma contamination**

All cell lines tested negative for mycoplasma

**Commonly misidentified lines**
(See ICLAC register)

N/A

## Animals and other research organisms

Policy information about studies involving animals; ARRIVE guidelines recommended for reporting animal research, and Sex and Gender in Research

**Laboratory animals**

Mus musculus. For ENU mutagenesis, male mice were used for the ENU injection and mated to female Th-MYCN (Tg(Th-MYCN) 41Waw) mice on an SvJ/129 background. All offspring were followed. For the backcrosses, C57BL/6 and Balb/c mice (C57BL/6JAusb and BALB/cJAusb) were used at mating age. All offspring homozygous for the MYCN transgene were followed. The Runx1t1 heterozygous knock-out mouse model (CBB6-Runx1t1tm1Fc/H) was imported and crossed to the Th-MYCN mice. All offspring were followed. For xenograft experiments, female NOD SCID GAMMA (NOD.Cg-Prkdc<scid>Il2rg<tm1Wjl>SzJAusb) mice, aged 5-6 weeks were injected with cells.

**Wild animals**

Study did not involve wild animals

**Reporting on sex**

For colony studies both male and female mice were used. For xenograft studies, only female mice were used.

**Field-collected samples**

No field collected samples

**Ethics oversight**

University of New South Wales Animal Care and Ethics Committee, Sydney Australia

Note that full information on the approval of the study protocol must also be provided in the manuscript.

# Clinical data

Policy information about clinical studies

All manuscripts should comply with the ICMJE guidelines for publication of clinical research and a completed CONSORT checklist must be included with all submissions.

| | |
|---|---|
| Clinical trial registration | N/A |
| Study protocol | N/A |
| Data collection | N/A |
| Outcomes | N/A |

# Plants

| | |
|---|---|
| Seed stocks | N/A |
| Novel plant genotypes | N/A |
| Authentication | N/A |

# ChIP-seq

## Data deposition

- [x] Confirm that both raw and final processed data have been deposited in a public database such as GEO.
- [x] Confirm that you have deposited or provided access to graph files (e.g. BED files) for the called peaks.

| | |
|---|---|
| Data access links<br>*May remain private before publication.* | To review GEO accession GSE230265:<br>Go to https://urldefense.com/v3/__https://www.ncbi.nlm.nih.gov/geo/query/acc.cgi?acc=GSE230265__;!!I9nncg!o-G42ac-PgnQeZPMk5G8ON-KSeqwg443HBH4axoVTcsWkKCXj6TQwn2F5eppgtrij97iERHQNHECLWimeanV6vA$<br>Enter the following token into the box: arulwewkxrehtuv<br>BED files containing replicate and consensus peaks can be found in https://github.com/CCI-BIO/RUNX1T1 |
| Files in database submission | GEO accession GSE230265 contains all raw sequencing (fastq), peak calling results (.xls) and associated coverage bigwig files (.bw) for each histone mark and condition.<br>https://github.com/CCI-BIO/RUNX1T1 contains BED files for consensus peaks from all replicates. |
| Genome browser session<br>(e.g. UCSC) | https://genome.ucsc.edu/s/njayatilleke/RUNX1T1_ChIPseq |

## Methodology

| | |
|---|---|
| Replicates | 3 technical replicates for each condition/treatment. 5 histone marks (H3K27ac, H3K27me3, H3K4me1, H3K4me2 and H3K4me3) for each of control and positive cells. |
| Sequencing depth | ChIP-seq was sequenced to a depth of 70M reads per sample and was output as single-end. |
| Antibodies | H3K27ac (Active motif #39133), H3K27me3 (Millipore #07-449), H3K4me1 (Active motif #39297), H3K4me2 (Active motif # 39141), H3K4me3 (Active Motif #39159) |
| Peak calling parameters | Bowtie2: -k 1, samtools: -q 30, MACS2: -p 1e-5 |
| Data quality | To ensure peak quality reads were aligned to a single position and filtered for quality using bowtie2 and samtools. Significance filtering was applied to the MACS2 output at a p-value of 0.0001 for narrow peaks and q-value 0.1 for broad peaks. Additional filtering for peaks of interest was applied downstream by observing fold-enrichment and q-values for overlapped peaks between replicates. |
| Software | Bowtie2 (v2.1.0), samtools (v1.9), MACS2 (v2.1.1), BEDTools (v2.26.0), R (v4.1.2), deeptools (v3.5.1). ChIP-seq analysis code can be found in the following public github repository: https://github.com/CCI-BIO/RUNX1T1 |
